# Global emergence and evolutionary dynamics of bluetongue virus

**DOI:** 10.1038/s41598-020-78673-9

**Published:** 2020-12-10

**Authors:** Moh A. Alkhamis, Cecilia Aguilar-Vega, Nicholas M. Fountain-Jones, Kai Lin, Andres M. Perez, José M. Sánchez-Vizcaíno

**Affiliations:** 1 Faculty of Public Heath, Health Sciences Centre, Kuwait University, Kuwait, Street 109, Jabriya Campus, P.O. Box 24923, 13110 Safat, Kuwait; 2 VISAVET Health Surveillance Centre and Animal Health Department, Veterinary School, Complutense University of Madrid, Madrid, Spain; 3 Department of Veterinary Population Medicine, College of Veterinary Medicine, University of Minnesota, St. Paul, USA; 4 School of Natural Sciences, University of Tasmania, Hobart, Australia

**Keywords:** Viral infection, Diseases, Virology, Evolutionary genetics, Molecular evolution

## Abstract

Bluetongue virus (BTV) epidemics are responsible for worldwide economic losses of up to US$ 3 billion. Understanding the global evolutionary epidemiology of BTV is critical in designing intervention programs. Here we employed phylodynamic models to quantify the evolutionary characteristics, spatiotemporal origins, and multi-host transmission dynamics of BTV across the globe. We inferred that goats are the ancestral hosts for BTV but are less likely to be important for cross-species transmission, sheep and cattle continue to be important for the transmission and maintenance of infection between other species. Our models pointed to China and India, countries with the highest population of goats, as the likely ancestral country for BTV emergence and dispersal worldwide over 1000 years ago. However, the increased diversification and dispersal of BTV coincided with the initiation of transcontinental livestock trade after the 1850s. Our analysis uncovered important epidemiological aspects of BTV that may guide future molecular surveillance of BTV.

## Introduction

Understanding the patterns of pathogen spread at a global scale is notoriously difficult. This is particularly the case for arthropod-borne multi-host viruses (hereafter arboviruses) that infect livestock. Often the reservoir species that are important for maintaining arbovirus prevalence in landscapes are unknown^1^ and epidemics are poorly sampled geographically making spread difficult to quantify. Outbreaks of arboviruses are not only a major public health threat but are often responsible for significant economic losses^2^. Effective molecular surveillance is crucial to characterize patterns of spread and facilitate pathogen control. For example, phylodynamic (ref) and phylogeographic models have provided fundamental insights into arbovirus spread across the globe^3^, yet for many arboviruses these models have yet been utilized.

Bluetongue virus (BTV) is one arbovirus that infects livestock worldwide and is responsible for global losses of agriculture of up to US$ 3 billion^2^. BTV causes severe infection in ruminants, especially sheep^4,5^. Domestic cattle have been implicated as an important reservoir for the disease^5,6^, yet wild ruminants has been also identified as a potential reservoir for BTV as well^4^, but the relative roles of each host species in BTV circulation remain unresolved. Midges (*Culicoides* spp.) are major vectors for the transmission of BTV between ruminants^7^, however instances of other modes of transmission, such as oral route, of BTV are possible^8,9^. In general, the geographical distribution of BTV serotypes worldwide is mostly determined by the location of distinct ecosystems of different midge species, including tropical and temperate regions^10,11^. BTV belongs to the family Reoviridae (genus *Orbivirus*) and has a double-stranded RNA genome consisting of 10 linear segments^12^. BTV is a rapidly evolving pathogen with a marked mutation rate ranging from 0.52 × 10^–4^ to 6.94 × 10^–4^ substitution rate per site per year for segments 2, 3, 6, and 10^13^. Segment 10 encodes non-structural protein NS3/NS3A and comprises approximately 800 nucleotides^14^. While segment 10 is the smallest among other gene segments, it was shown to be suitable for Bayesian phylogeographic analyses^13^. Currently, BTV is classified into 27 antigenically distinct serotypes^15^. Serotypes 1–24 are likely to be predominantly transmitted by midges^16^, while there are uncertainties around the role of midges in the transmission of serotypes 25 and 26^17,18^. BTV control and eradication is heavily dependent on vaccination and movement restriction. However, the use of BTV live attenuated vaccines is a consequential anthropological factor for the dissemination of the disease on an intercontinental scale^19^.

BTV was first described in South Africa in 1876 when European sheep intensive farming was introduced in the region^16,20^. However, the vast geographical expansion of vector range and animal movement through trade led to the subsequent spread of BTV throughout Africa^21^. In 1924, BTV outbreaks were observed among sheep in Cyprus^21^. Subsequently, after 1950 the disease has been identified in Asia, Europe, Australia, and North America and thus, has been recognized as an emerging transcontinental disease^16,21^. The disease became endemic in many regions worldwide, where it either causes outbreaks through the year in tropical countries or distinctly seasonal outbreaks in temperate countries^16^. Beside animal movements, climate change has been recognized as the major factor for the transformation of BTV global distribution and epidemiology^16,22,23^. Subsequently, the geographical distribution of BTV outbreaks is also shifting especially in Europe, where exotic and novel strains have been introduced from multiple continents^24–26^. During the 2000s, BTV moved from southern to northern Europe causing severe outbreaks and unprecedented economic losses, mostly by serotypes 1, 4 and 8^2,15,23^. Over 15 serotypes and 108 novel strains have been reported in India and China, making both countries also of potential importance for BTV emergence and spread^27–29^. However, very few studies have attempted to reconstruct the evolutionary history of BTV on either local or regional scales^13,30–32^. Given the continuous emergence of novel BTV strains and the subsequent impact of their outbreaks on livestock populations worldwide, especially Europe, exploring the global evolutionary epidemiology of BTV is a critical element for designing risk-based intervention programs, such as identifying and targeting areas infected with emerging strains.

Here we applied Bayesian phylodynamic methods to reveal the global spatiotemporal and cross-species characteristics of BTV using a commonly sequenced segment in an integrated statistical framework. Phylodynamic methods have become well-established and have been shown to be the most robust tool for tracing historical origins and transmission of rapidly evolving pathogens like BTV^33,34^. The objective of our study was to infer geographical origins, intercontinental spread, significant dispersal routes, and reservoir dynamics of BTV using phylodynamic analyses on all publicly available segments 10 (S10) and 6 (S6) gene sequences. We reveal new evolutionary insights into BTV spread and transmission cycles between hosts across the globe. Such findings may be used to inform and enhance current molecular surveillance efforts of BTV worldwide and subsequently reduce outbreaks caused by both emerging and endemic strains.

## Results

### Demographic history

**Figure 1:**
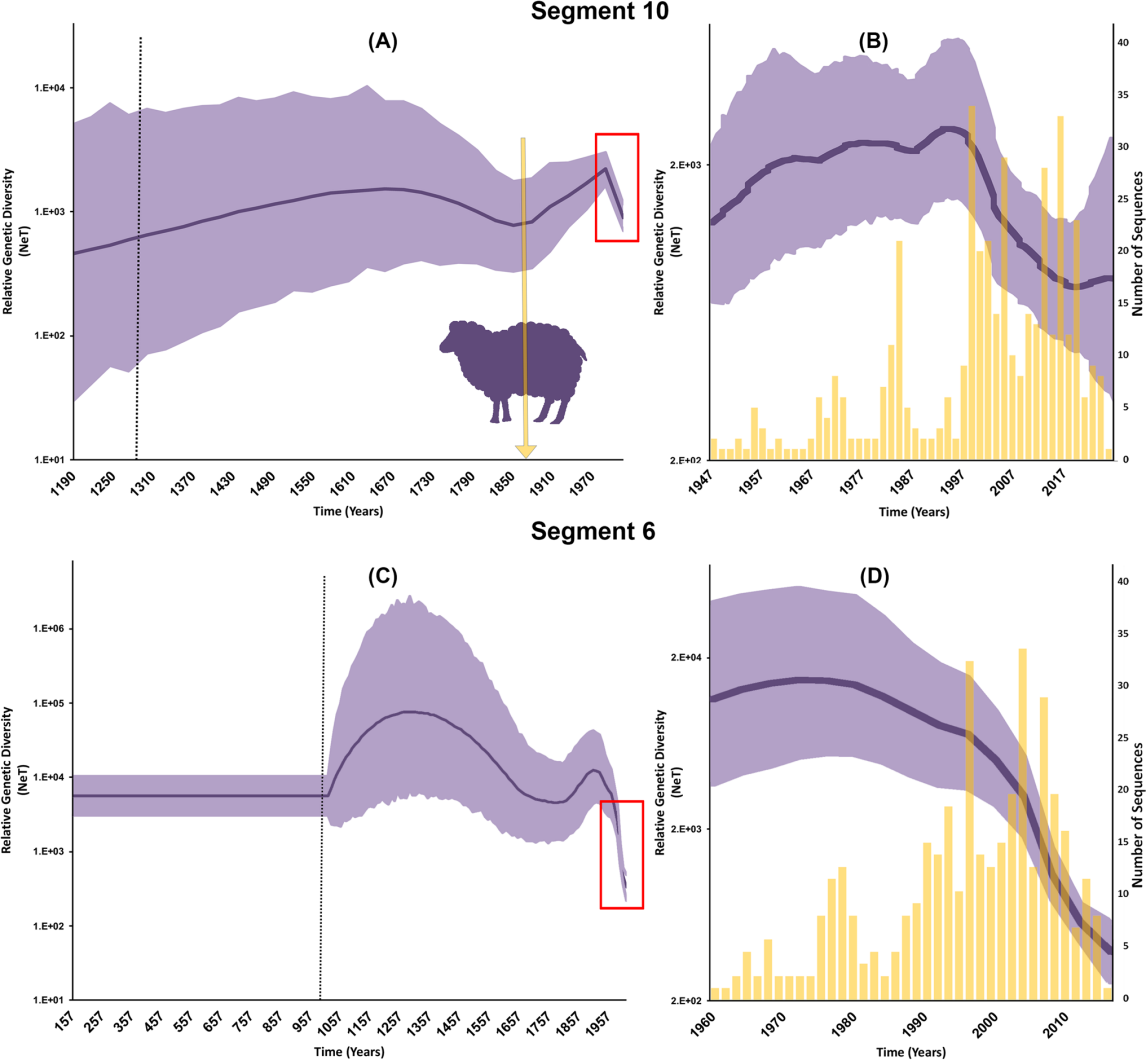
Bayesian Skygrid (BSg) plots of the relative genetic diversity through time segments 10 and 6 sequences of Bluetongue virus between 1958 and 2016. The posterior median estimate is indicated by the dark purple line, and the light purple shaded areas correspond to the 95% high posterior density (HPD). (**A**,**C**) Vertical dotted line represents the estimated time at which the relative genetic diversity transitioned from a slow to a fast growth rates, the red rectangle highlights the genetic diversity of the present sequence data. The yellow arrow indicates the time when Merino sheep farming was introduced from Europe to Africa. (**B**,**D**) BSg plot is a detailed magnification for genetic diversity of the sequences included in the BEAST analyses, and the yellow bars indicate their temporal distribution. (**A**,**B**) Indicate segment 10. (**C**,**D**) Indicate segment 6.

**Figure 2:**
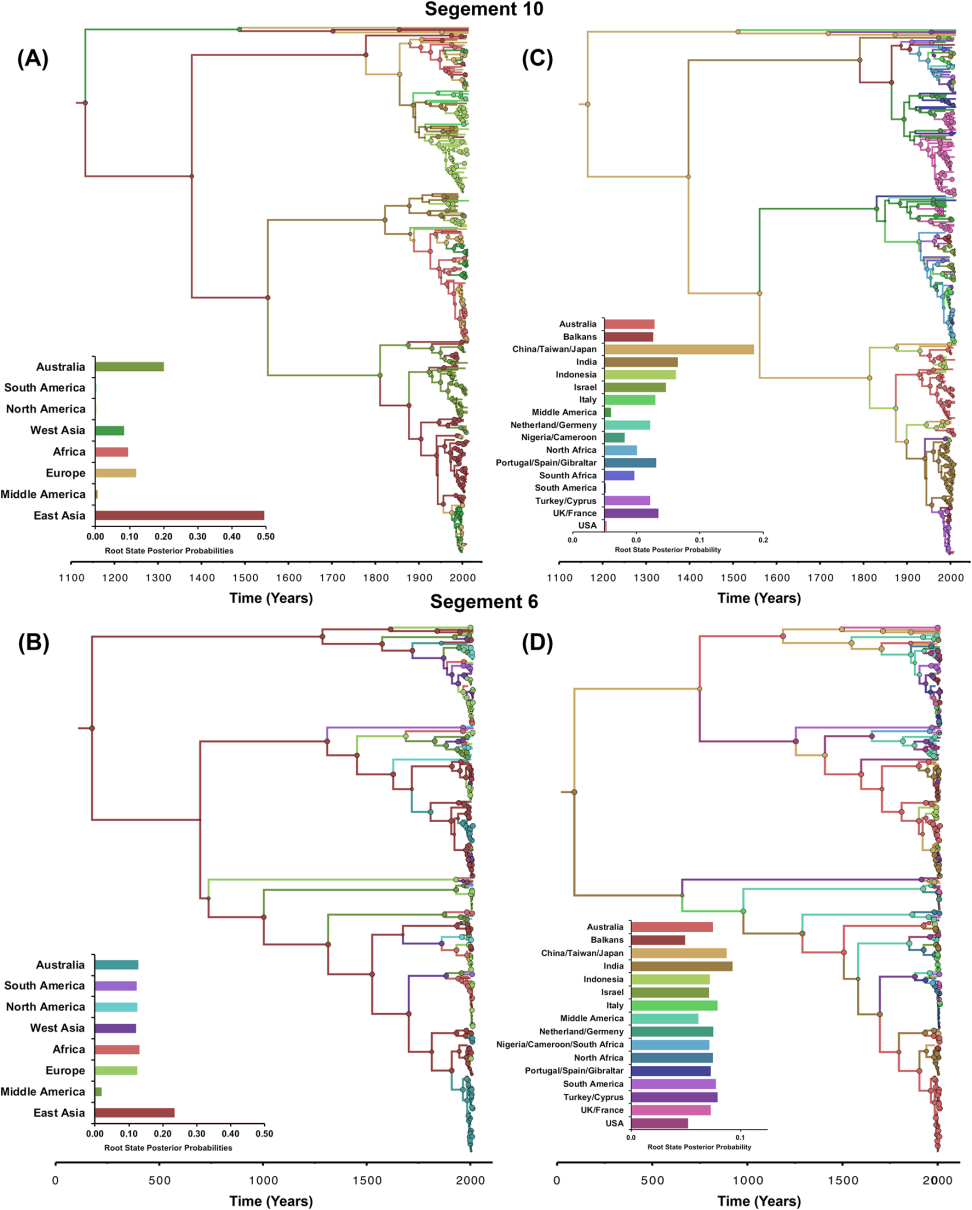
Maximum clade credibility (MCC) phylogeny of segments 10 and 6 sequences of Bluetongue virus between 1958 and 2016. The trees are inferred from the symmetric phylogeographic model with reversible transitions. Colour of the branches represents the most probable location state of their descendent nodes. The color-coding corresponds to the upper left bar chart which represents the root location state posterior probabilities for each country. Circle sizes are proportional to the posterior node support. (**A**,**B**) MCC tree inferred from continental level phylogeographic analysis (**C**,**D**) MCC tree inferred from country level phylogeographic analysis. (**A**,**C**) indicate segment 10. (**B**,**D**) indicate segment 6.

Our demographic reconstruction of BTV through time revealed that the approximate divergence time of the virus was inferred around 1000 years ago (Figs. 1A, 2A,C) using the S10 gene segment. However, when using the S6 gene, we inferred a deeper divergence time of around 2000 years (Figs. 1C, 2A,C). Both gene segments showed a remarkable increase in the genetic diversity during the beginning of the second millennia, followed by a drastic decline till the late 1700s (Fig. 1A,C). Genetic diversity of S10 increased after the 1850s (Fig. 1A) and peaked in the 1980s (Fig. 1B). Yet, genetic diversity of S6 peaked in the 1850s (Fig. 1C), then drastically declined (Fig. 1D). In contrast, our phylodynamic model indicated a substantial decline in genetic diversity of S10 from 1990 until 2010, followed by a slight increase from 2011 till 2016 (Fig. 1B). For both segments, the BF comparisons of marginal likelihoods estimated by the stepping-stone sampling and path-sampling methods significantly favored the exponential growth coalescent tree model with the uncorrelated lognormal branch-rate prior (BFs > 4; see Supplementary Tables S5 and S6). The inferred posterior estimate of the mean nucleotide substitution rate for S10 was equal to 4.26 × 10^–4^/site/year (95% highest posterior density (HPD): 3.38 × 10^–4^–5.09 × 10^–4^), while for S6 was 5.02 × 10^–4^/site/year (95% highest posterior density (HPD): 4.42 × 10^–4^–6.71 × 10^–4^).

### Phylogeographic history

The results of the global phylogeographic analyses suggest that BTV emerged from East Asia (root state posterior probability (RSPP) = 0.48 and 0.23 for S10 and S6, respectively; Fig. 2A,B), specifically from China and India over 1000 years ago (RSPP = 0.19 for S10 and 0.074 for S6; Fig. 2C,D). Bayes factor (BF) comparisons revealed the symmetric phylogeographic model with reversible transitions was the best fitting model for both S10 and S6 (BF > 10). Using the S10 gene, we inferred that viral migrations between Asia and Australia started in the mid-1500s, while migrations between Asia and Europe started in the late 1700s. Further, migrations between Europe and Africa were initiated in the mid of 1800s in line with the increase in diversity at these periods with many emerging new viruses migrating between the continents in the 1950s (Fig. 2A). Our analysis provides evidence for the Balkans region as the portal of BTV introduction to Europe in the Mid-1700s. We also detected potential transcontinental migration of BTV from Middle America to the USA and South America between the 1800s and the 1900s (Fig. 2C). Additional notable incursions within the same period were inferred from Indonesia to Australia and India, originating from China (Fig. 2C). Similarly, using the S6 gene, we inferred that East Asia was the epicenter of the transcontinental spread (Fig. 2B), mainly from India and China (Fig. 2D), but with deeper root in time (i.e., over 1500 years ago).

**Figure 3:**
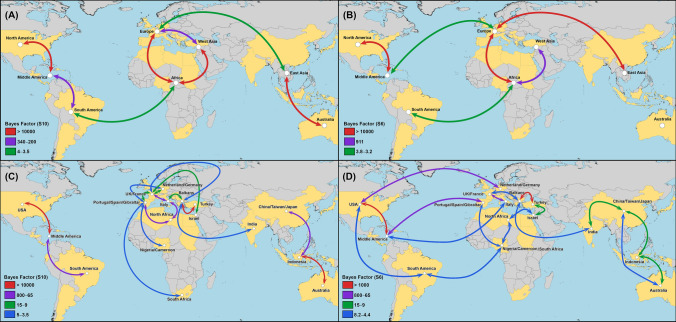
Global exchange routes of bluetongue virus. Bayesian stochastic search variable selection (BSSVS) was used to infer dispersal routes with none-zero rates using Bayes factors. Dispersal routes that were inferred to have significant support (Bayes Factor > 10) are plotted. Red arrows represent migration routes with the strongest statistical support (BF > 2000) as indicated by the legend on the lower left. (**A**,**B**) Dispersal routes inferred from continental level phylogeographic analysis (**C**,**D**) Dispersal routes inferred from country level phylogeographic analysis. (**A**,**C**) indicate segment 10. (**B**,**D**) indicate segment 6. Maps were generated using SPREAD3 version 0.96 (https://rega.kuleuven.be/cev/ecv/software/SpreaD3).

The Bayesian stochastic search variable selection (BSSVS) approach suggested significant non-directional dispersal routes for BTV between Asia, Africa, Europe, Australia, Middle, South, and North America (Fig. 3A). Most significant bidirectional dispersal routes were detected between North and Middle America, Africa, Europe, and West Asia, as well as East Asia and Australia (BSSVS-BF > 10,000; Fig. 3). However, transcontinental dispersal routes between East Asia and Europe, as well as between Africa and South America, were the least significant among other dispersal routes (BSSVS-BF < 5; Fig. 3A). We found similar results using the S6 gene but with the absence of significant migration routes between Middle and South America, East Asia, Australia, and West Asia and Europe (Fig. 3B). Country-level BSSVS-BF using S10 gene results indicate that the most significant viral dispersal routes were between the USA and Middle America, the Balkans region, Turkey, and Indonesia, and Australia (Fig. 3C). A prominent circle of dispersal routes was inferred between Italy, the North African region, and France (BSSVS-BF > 65; Fig. 3C). Similarly, using the S6 gene, we inferred that the migration routes between Turkey and the Balkans, as well as between Middle and North America, were the most significant among other routes (BSSVS-BF > 1000; Fig. 3D). Furthermore, results revealed significant migration routes between European countries on one side and North and Middle America on the other (BSSVS > 65; Fig. 3D). Nevertheless, unlike inferences from the S10 gene, the S6 gene revealed less significant migration routes between East Asian countries and Australia (BSSVS-BF < 65; Fig. 3D).

**Figure 4:**
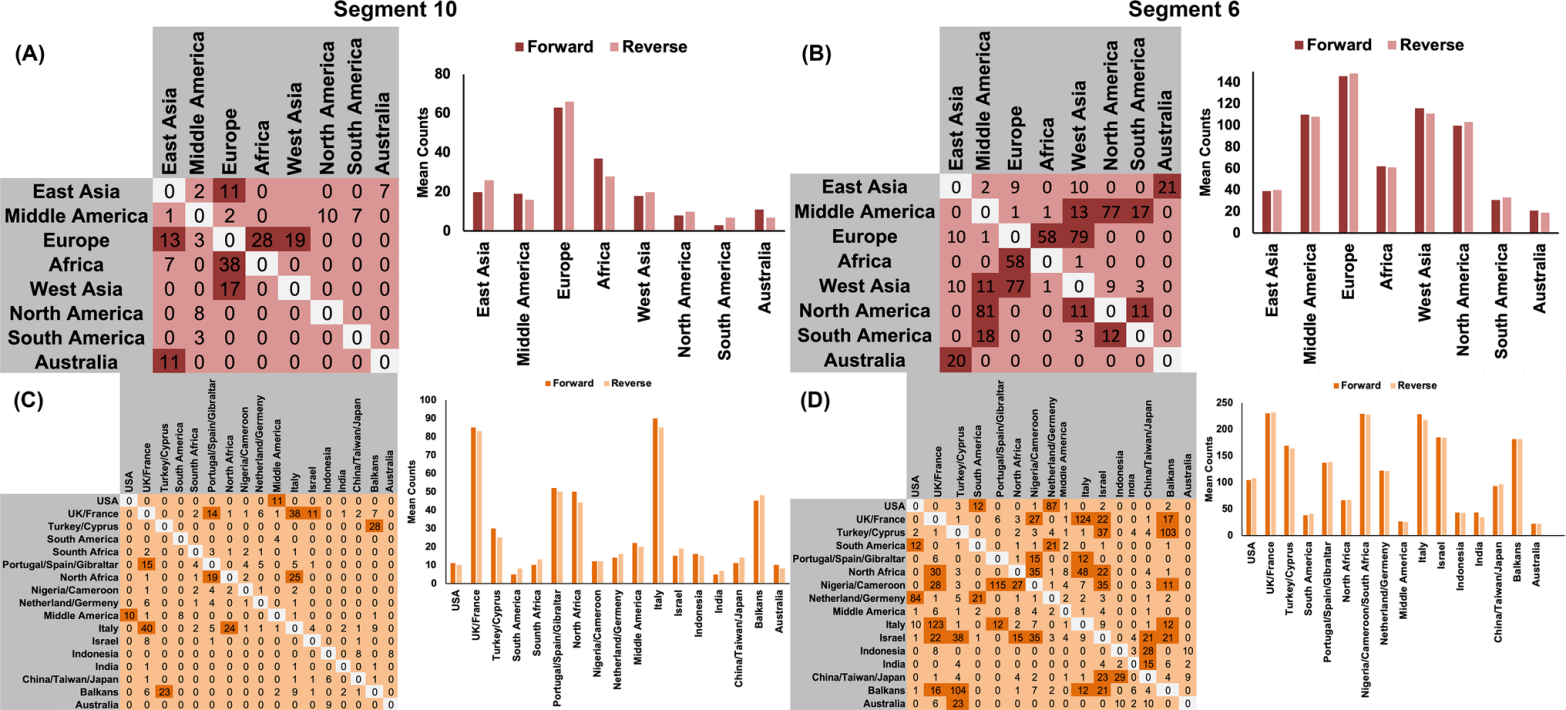
Global posterior mean counts of the Markov jumps (MJ) over the phylogeny of bluetongue virus. Matrix on the top summarize relative forward (columns) and reverse (rows) transitions between pairs of discrete traits (i.e., countries). Cells shaded dark orange indicate MJs greater than ten. Charts on the bottom summarize total relative forward and reverse transitions for each country. (**A**,**B**) MJ estimates inferred from continental level phylogeographic analysis (**C**,**D**) MJ estimates inferred from country level phylogeographic analysis. (**A**,**C**) Indicate segment 10. (**B**,**D**) Indicate segment 6.

The results of both gene segments agree on Europe having the highest forward and reverse transitions in terms of viral jumps between continents (Fig. 4A,B). Yet, while the S10 gene indicates that the most intense viral jumps were between Europe and Africa (Fig. 4A), S6 indicate more intense viral jumps between Europe and West Asia (Fig. 4B). Results of both gene segments also show that the lowest number of viral jumps inferred for South America (Fig. 4A,B). Country-level results of both gene segments suggest that the dispersal route, which experienced the highest number of viral jumps, was from Italy to UK/France and vice versa (Fig. 4C,D). Additionally, the forward and reverse transitions between countries were highest for France and Italy (Fig. 4C,D).

### History of cross-species transmission

**Figure 5:**
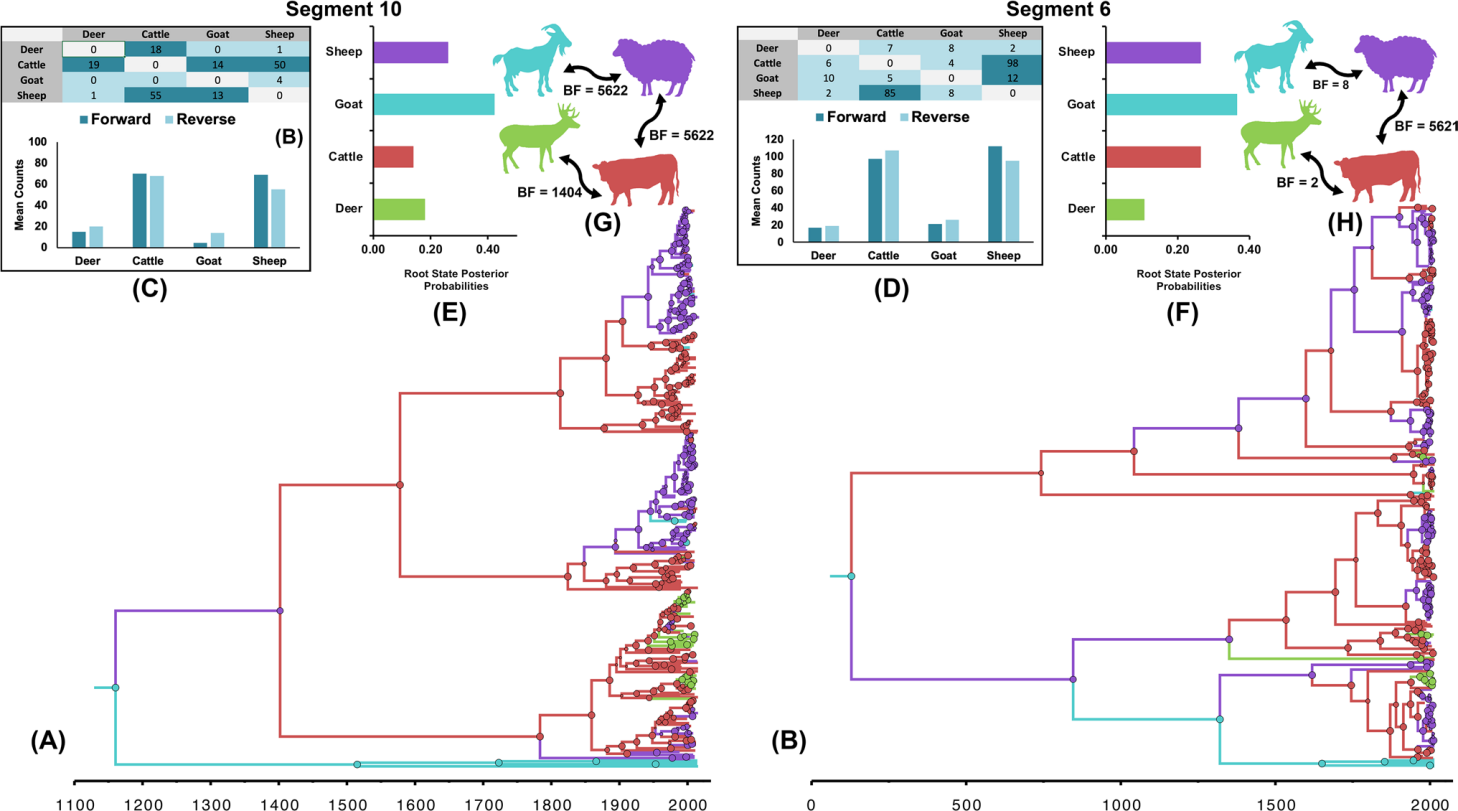
Ancestral host-species discrete trait reconstruction for segments 10 and 6 sequences of Bluetongue virus between 1958 and 2016. (**A**,**B**) Maximum clade credibility (MCC) tree. Branches’ colours represent the most probable host state of their descendent nodes and color-coding corresponds to (**E**) and (**F**). (**C**,**D**) Posterior mean counts of the Markov jumps (MJ) over the phylogeny of bluetongue virus. Matrix on the top summarize relative forward and reverse transitions between pairs of discrete traits (i.e., hosts). (**E**,**F**) Root state posterior probabilities (RSPP) inferred from the symmetric host discrete trait model. (**H**,**I**) Significant transmission routes between host species inferred BSSVS approach. BF values are on the top of the arrows. Blue indicates goat, purple indicate sheep, red indicate cattle, and green indicate deer. Cells shaded dark blue indicate MJs greater than 10. Charts on the bottom summarize total relative forward and reverse transitions for each host. (**A**,**C**,**E**,**G**) Indicate segment 10. (**B**,**D**,**F**,**H**) Indicate segment 6.

Using both gene segments, we found strong evidence that goats were the likely ancestral host of the virus (RSPP = 0.43 for S10 and 0.36 for and S6; Fig. 5A,B,E,F). However, the early transmission of the virus between goat and sheep may have resulted in a subsequent explosive emergence of new trains transmitted from sheep to cattle, then to other species, which continued until recently (Fig. 5). Like the results of the phylogeographic model described above, our sequence data favored the symmetric phylodynamic model (BF > 34) when using host status as a discrete trait. Results from S10 gene indicate that the highest significant bidirectional transmission routes were between sheep-goat and sheep- + cattle (BSSVS-BF = 5622; Fig. 5G). This was followed by a transmission route between cattle and wild ungulates (BSSVS-BF = 1404; Fig. 5G). Yet, when using inferences from S6 gene, transmission routes between goat and sheep as well as cattle and deer become substantially less significant (BSSVS-BF < 10; Fig. 5H). We found no statistically significant transmission route for either gene region between cattle and goats or between wild ungulates and sheep and goats (Fig. 5G,H). Complementing this analysis, our Markov Jump analysis identified that the most intense route inferred between sheep and cattle for both gene regions (Markov-jump (MJ)s ≥ 50; Fig. 5G,H). We estimated moderate number of viral jumps from goats to sheep (MJ = 13 and 8 for S10 and S6, respectively; Fig. 5G,H), yet there were very few viral jumps inferred from goat to other species. The statistically significant p-values (< 0.01) of the Ai and Ps scores suggest that our selected discrete traits for country and host species did contribute to the structure of the posterior evolutionary tree of BTV (see Supplementary Table S7).

## Discussion

Using an integrated Bayesian phylodynamic methodology we uncovered new insights into the global evolutionary epidemiology of BTV. We shed novel insights into the phylogeographic history of virus origins and dispersal between countries worldwide and quantified its transmission dynamics between hosts and vectors. These insights can not only inform future BTV molecular surveillance efforts worldwide but assist with control efforts on this economically important pathogen.

We estimated that BTV has circulated in ungulate populations for at least 1000 years based on the S10 gene and potentially more than 2000 years based on the S6 analysis (Fig. 2A). These estimates are in the range of previous work^13,30,31^ and are supported by older origins in line with the expansion of trade between the east and the west through the silk rood^35^. It is worth noting that the S6 gene exhibited a substantially slower rate of evolution than the S10 gene, therefore it is expected to have deeper origins in time. Both gene segments demonstrated similar increasing patterns in the genetic diversity during the beginning of the second millennium, that was followed by a notable decline, but with a substantial amount of uncertainty (Fig. 1A,C). Yet, Genetic diversity through time in our study demonstrated that the reestablishment of BTV’s exponential population growth after the 1850s coincided with the increase of animal trade activities between continents (Fig. 1A,C), such as the introduction of Merino sheep farming from Europe to Africa^20,21^. Interestingly, the genetic diversity of BTV continued to peak in the 1900s, even with the introduction of live attenuated vaccines in 1906 (Fig. 1B)^36^. The period between the 1900s and 1980s was dominated by the use of live attenuated and inactivated vaccines, which had inconsistent results of success and failure^37^. However, the introduction of recombinant vaccines in the 1990s led to a substantial decline in the genetic diversity of BTV worldwide (Fig. 1B; Refs.^37,38^). Indeed, during the past three decades, a whole new-generation of BTV vaccines were introduced and were shown to be more effective in terms of providing a protective immune response and cross-protective against more than serotype^39^. That said, the continuous emergence of new strains worldwide indicates that vaccine development barely keeps up with the rapid BTV reassortment^40^.

Our phylogeographic model (Fig. 2) suggests that East Asia, particularly China and India, are the ancestral location of origin and dissemination of BTV worldwide (Fig. 2C,D), where many novel serotypes and strains are continuously reported^29,41–44^. While our inference contradicts the common notion that BTV emerged from Africa^45^, several environmental risk factors for BTV outbreaks have been identified in China and India, including the abundance of specific species of midge and climatic conditions which made it a suitable geographical area for emergence, spread, and maintenance of new viruses^28,42,46^. Our phylogeographic analyses (Fig. 2A,B) provide further evidence for the importance of the East Asian region as a hotspot for BTV. In contrast, European BTVs originated from Asia (both East and West) with the Balkans region (specifically Greece) as the main portal of entry and spread (Fig. 2). These results continue to support the notion that the Balkans region is an essential ecological niche for BTV incursions into Europe^47^. However, our results suggest that Europe is potentially the primary source of BTV introductions into Africa and Latin America (Fig. 2A,B). Indeed, the colonization activities of both continents by Europe was accompanied by intensive exportation of livestock between the 1700s and 1800s. Consequently, our results implicate Latin America as the source of novel BTV introductions into North America in the 1900s (Fig. 2). These novel incursions were attributed to windborne midges carrying BTVs from endemic regions in Latin America, such as the Caribbean Basin, to south-eastern USA^48,49^.

We found little evidence of directionality of BTV dispersal routes between countries (BF < 10; Fig. 3). This indicates a homogenous spread process of BTV between pairs of geographical locations, which might be maintained by the broad geographical distribution of *Culicoides* spp.^50^. The strongly supported dispersal route (BF-BSSVS > 10,000) inferred between Indonesia and Australia (Fig. 3C) confirms the notion that BTV dispersers between northern Australia and Indonesia characterized by higher genetic diversity than isolates collected from eastern Australia, as described elsewhere^51^. In addition, our results indicated that Indonesia is an important portal for virus exchange between China and Australia (Fig. 3A,C,D)^41^. The other three statistically significant dispersal routes were inferred between Middle and North America, West Asia and Africa, and Europe and Africa (Fig. 3A,B). However, the route between Europe and Africa was the most intense in terms of viral jumps (Fig. 4A,B), which was also maintained by notable migrations with north, mid and south African countries (Fig. 3C,D). Indeed, our results revealed a circle of significant and intense dispersal routes between countries surrounding the Mediterranean Basin (Fig. 3C,D), which is attributed to the hot and dry climatic conditions as well as the abundance of multiple *Culicoides* spp^24,52–54^. These factors seem to have significant impacts on shaping the evolutionary characteristics of endemic and emerging BTVs. Further, animal movements are restricted in Europe, and therefore the statistically significant inferred dispersal routes reflect the importance of vector involvement in the transmission and maintenance of BTV between countries^50^. Additionally, within Europe, altitude, wind, and livestock densities have been found to predict the spread of BTV in Europe^32^ and these factors are also associated with *Culicoides* spp dispersal which supports our findings in term of inferring the highest number of significant dispersal routes on global scales (Fig. 3B). However, it worth noting the dispersal route between Turkey and the Balkans also represents an essential introductory route for new BTVs into Europe (Fig. 3C,D). Our results indicate that transcontinental dispersal of BTV between the Western and Eastern hemispheres, at least in the short term, plays less of a role in the emergence and spread of recent outbreaks. Moreover, the reduced role of intercontinental spread may be evidence for the role of current global trade restrictions from infected countries that has shaped the genetic evolution and diffusion of BTV.

While sheep are the most affected host species from BTV infections^5^, our analyses suggest that goats are the ancestral host for the virus (Fig. 5A,B,E,F). Goats have always been recognized as major hosts for BTV^55–59^. The extended period of viremia of BTV infections in goats^18,60^ makes this species an ideal reservoir for newly emerging BTVs by providing the perfect conditions for continuous genetic reassortment processes^59^. Furthermore, goat density was also implicated in the evolution and spread of BTV outbreaks in Europe^32^. China and India have the largest goat populations worldwide^61^, which may explain why China was inferred as the ancestral country of BTV origins and dispersal using our selected phylodynamic model (Figs. 2, 5). Our analyses, however, implicate sheep and cattle as the responsible host for the later increase in the genetic diversity over time (Fig. 5A,B). This reflects the high magnitude of BTV pathogenicity on sheep in terms of the severity of clinical symptoms, morbidities, and mortalities^5^, while highlights the role of cattle as the primary reservoir for the virus^4,6^. Also, cattle may be a preferred target species for biting midges as they are the largest species commonly infected by BTV, often lack a wooly coat and the duration of their clinically detectable viremia is longer than in sheep^62^. Additionally, the intensity of forward and backward viral jumps among species was the highest between sheep and cattle (MJ S10 = 55 and 50, MJ S6 = 85 and 98, respectively; Fig. 5C). This intense transmission route between both species seems to be essential for maintaining the genetic mutation processes of BTV which subsequently lead to the frequent emergence of new viruses (Fig. 5D)^63^. There was a strong and exclusive transmission cycle between sheep and goats (Fig. 5G,H). which is not surprising since sheep and goats are often raised in the same farms or production systems. Furthermore, our modeled transmission cycle demonstrates the importance of cattle also as a central reservoir for the transmission of BTV between hosts (Fig. 5G,H). Even though wild ungulates, such as white-tailed deer, can suffer from apparent clinical infection, our results indicate that the role of wild ungulates as a reservoir is minimal in shaping the global evolutionary epidemiology of BTV (Fig. 5).

Our study has several weaknesses that are typical to most phylodynamic studies due to the inherent nature of the available sequences for animal pathogens, specifically those retrieved from surveillance data. One important weakness is that our phylodynamic models were constructed using S10 and S6 gene segments, and the evolutionary history of other gene segments was ignored. However, both segments mostly agreed on the global evolutionary history of BTV and were shown to be appropriate for the discrete trait analyses (i.e., based on statistical significance of the Ai and Ps score; see Supplementary Table S7), when compared to other gene segments^13^. There is a common notion that advocates for using phylodynamic methods on full genome sequences to provide a deeper resolution on the evolutionary history of rapidly evolving pathogens like BTV. However, such a strategy is ill-timed, requires massive computational resources, and leads to discarding a large number of sequences due to the rapid reassorting nature of BTV. Further, single gene segments from other rapidly evolving pathogens like HIV, which have similar evolutionary characteristics, were used to infer its global evolutionary epidemiology, as described elsewhere^64^. Hence, full genome analyses of BTV is inefficient for targeted or near-real-time molecular surveillance systems worldwide. Yet, future studies should attempt to further explore the evolutionary history of BTV using either full genome or other gene segments, when sufficient computation resources are available. That said, our phylodynamic analyses was based on all available S10 and S6 gene sequences collected by passive or active surveillance and associated with BTV epidemics observed worldwide. However, the reassorting nature of BTV did make us lose more than 26% of the unique sequences, and subsequently, their metadata related to geography and host. Therefore, while our inferences have biological plausibility, they might not reflect the accurate evolutionary history of BTV. Yet, our analyses were completely insensitive to unequal number of sequences obtained from different countries and hosts (Fig. 2C,D; see Supplementary Tables 3, 4). For example, our Skygrid results (Fig. 2B,D) showed a decline in genetic diversity even though a greater number of sequences were collected in later years. In addition, we inferred China (n for S10 = 7, and n for S6 = 8) as the ancestral country of origin (Fig. 3) and goats (n for S10 = 13, and n for S6 = 5) as the ancestral host (Fig. 5C), while substantially more sequences were collected from the USA, sheep and cattle, (see Supplementary Tables 3, 4). Further, we randomly reintroduced and removed laboratory strains into our analyses to test the sensitivity of the inferred posterior estimates, and we found no notable changes in the major results that could affect our interpretations. Finally, most of Africa remains either unsampled or under sampled (Supplementary Table S3) for bluetongue where outbreaks are reported^65^ and therefore is underrepresented in our phylodynamic analyses. Thus, our present study provides an important justification for establishing a targeted molecular surveillance program in affected African countries.

Our study provided unique insights into the global evolutionary epidemiology of BTV, where we highlighted historical dispersal routes of the virus between and within continents worldwide and its transmission cycle between hosts. Results indicate the genetic diversity of BTV re-peaked between the 1930s and the 1980s followed by drastic decline with the development of new vaccination regiments. We identified China and India, countries with the highest population of goats, as the ancestral country for BTV emergence and dispersal worldwide over 1000 years ago. However, significant diversification and dispersal events of BTV coincided with the initiation of transcontinental livestock trade after the 1850s. Additionally, the Balkans region remains an essential ecological niche for BTV incursions from Asia into Europe. Further, our results revealed a notable circle of significant and intense dispersal routes between countries surrounding the Mediterranean Basin, which is attributed to the hot and dry climatic conditions as well as the abundance of multiple *Culicoides* spp. We inferred that goats are the ancestral hosts for BTV, while sheep played the most significant transmission role among other species. Finally, our results indicate that cattle continue to be the most important reservoirs for BTV, while wild ungulates have substantially less role in the transmission of the disease. These results provided new comprehensive and quantitative insights into the global epidemiology of BTV, which subsequently can be used to guide current BTV molecular surveillance and intervention strategies worldwide.

## Methods

### Data sources and preliminary phylogenetic analyses

We retrieved all complete and near complete S10 and S6 gene sequences of BTV isolated worldwide between 1937 and 2016 from GeneBank. We also retrieved associated metadata, which included, isolate name, collection date, country of origin, isolate type, and host species. Additional sequence metadata were retrieved from the related published literature and the online phylogenetic sequence analysis and improved diagnostic assay system for viruses of the family *Reoviridae* (ReoID) library^66^. The final data comprised a total of 700 and 559 sequences for S10 and S6 gene segments, respectively, collected from 52 countries worldwide. We used year of collection to estimate divergence times, and therefore, sequences with no time information were excluded from the analyses [n = 122(17.3%) for S10; n = 51 (9%) for S6]. Vaccine and laboratory strains were also excluded from the sequence dataset [n = 18 (2.6%) for S10; n = 101 (18.1%) for S6].

We used AliView version 1.18^67^ and MUSCLE version 3.8.3^68^ to align and confirm the reading frame by examining the amino-acid translation of 558 S10 and 487 sequences. The length of the final alignment of the S10 sequences ranged from 690 to 822 bp, and 1572–1638 bp for S6. We mapped the geographical distribution of the sequences and used the GTR + Γ substitution model implemented in RAxML version 8.0^69^ to assess their phylogenetic tree topology (see Supplementary Fig. S1). We found no recombination events in the sequence data using Recombination Detection Program version 4^70^. We removed 100% identical sequences from the final dataset, and therefore, leading to a total of 389 and 337 field strains for S10 and S6 genes, respectively, included in the subsequent phylodynamic analyses (see Supplementary Tables S1 and S2)^71^. Finally, we used TempEst version 1.5.3 to assess the strength of the temporal signal in the sequences dataset as well as identify sequences with incongruent genetic divergence and sampling dates^72^.

### Reconstruction of the virus demographic history

We used the relaxed molecular-clock models implemented in BEAST version 1.10.4^73^ to reconstruct BTV’s demographic history and infer divergence times within a Bayesian analytical framework. We selected the non-partitioned GTR + Γ substitution model for our multi-sequence alignment using Bayesian information criterion^74^ estimated by PartitionFinder version 2.1.1^75^. We evaluated three parametric and one non-parametric coalescent node-age models to reconstruct BTV’s historical population growth through time and its estimate divergence time. Our selected parametric priors included the constant population size^76^, the expansion growth^77^, and the exponential growth^77^, while the non-parametric coalescent prior was the Bayesian Skygrid^78^. Furthermore, we assessed the uncorrelated lognormal and exponential branch-rate priors for each coalescent tree model. Therefore, we compared eight candidate relaxed-clock models using a Bayes factor (BF) approach in order to identify the best fitting demographic model for our dataset. We used the stepping-stone sampling and path-sampling methods^79^ to estimate the marginal-likelihood of each candidate model. We then used the BF comparisons from the resulting marginal-likelihood estimates to select among the relaxed-clock models.

We used Bayesian Markov chain Monte Carlo (MCMC) simulations for 300 million cycles and sampled every 3000th state to infer the posterior evolutionary parameters of BTV’s phylogeny under each candidate model. We used duplicate MCMC runs for each model to assess the stability of their marginal-likelihoods estimates. Convergence of the posterior parameters was assessed through effective sample size (i.e., ESS > 200) using Tracer version 1.7.1^80^. For the S6 gene, we had to combine three MCMC simulations with a total of 900 million cycles using LogCombiner to achieve model convergence. We used TreeAnotator version 1.10.4 to discard the first 10% of the samples from each MCMC chain and summarized the resulting posterior probability density as a maximum clade credible (MCC) tree. We used tracer to generate plots for the estimates of the effective population over time from the Bayesian Skygrid coalescent tree with the uncorrelated lognormal branch-rate models.

### Discrete phylogeography and transmission between hosts

We inferred the global geographic origins of BTV and its significant dispersal routes between infected countries using discrete-state ancestral reconstruction methods implemented in BEAST^81^. To assess the impact of the spatial trait resolution on the inferred phylogeographic history, we specified a total of 8 and 17 discrete traits to model spatio-temporal diffusion of BTV between continents and countries, respectively (see Supplementary Tables S3 and S4). The continental level phylogeographic analysis included North America, Middle America, South America, Europe, Africa, West Asia, East Asia, and Australia. While, for the country-level analysis, we aggregated countries with few sequences with its neighboring countries to form distinct geographical regions, in order to reduce the impact of biased posterior inferences resulting from discrete traits with very few sequences^82^. However, countries with sufficiently large number of sequences and notable BTV outbreaks were assigned as an independent spatial discrete trait. Thus, the aggregated geographic areas, included, Balkans (compromised Bosnia and Herzegovina, Bulgaria, Greece, Hungary, and Kosovo), China (aggregated with Taiwan and Japan), Middle America (including Barbados, Costa Rica, Dominican Republic, Guatemala, Honduras, Jamaica, Martinique, Panama, Puerto Rico, and Salvador), Netherland (aggregated with Germany), Nigeria (aggregated with Cameroon), North Africa (compromised Algeria, Egypt, Libya, Morocco, Sudan, and Tunisia), Spain (aggregated with Portugal and Gibraltar), South America (compromised Argentina, Brazil, Ecuador, Guyane, and Trinidad and Tobago), Turkey (aggregated with Cyprus), and France (aggregated with the UK), while non-aggregated countries included India, Israel, Italy, Australia, South Africa, and the USA. We aggregated South Africa with Nigeria and Cameroon for the S6 region as only one sequence from each country was available (see Supplementary Table S4).

To model the transmission between host species, we used sheep (*Ovis aries*), goats (*Capra aegagrus hircus*), cattle (*Bos taurus*), and deer (family Cervidae; wild ungulates) as discrete traits. The best fitting coalescent tree model and branch-rate prior combinations to the sequences data, selected as described above, was used for the subsequent ancestral-trait reconstruction analyses. We used the Bayesian stochastic search variable selection (BSSVS) method, implemented in BEAST, to identify significant migration routes and their directionality between countries and hosts. Also, we assessed the fit of the sequence data to two candidate discrete-trait models, namely the symmetric model (reversible transitions) and the asymmetric model (irreversible transitions) using the BF comparisons, as described above. Using the Markov-jump (MJ) method^83^, we inferred the intensity of backward and forward transitions within discrete trait matrices, as a proxy for the mean number of viral jumps between countries or hosts.

We used FigTree version 1.4.4^84^ to summarize the root state posterior probabilities (RSPP) of the discrete traits at the internal nodes of the MCC trees inferred from the selected phylodynamic models. SPREAD3 version 0.9.6 was used to assess the statistical significance (BF > 3) of viral dispersal routes between countries and hosts. The spatio-temporal evolutionary history of the MCC tree and significant dispersal routes between countries were mapped using JavaScript Object Notation (JSON) maps and SPREAD3 version 0.96^85^. Finally, we assessed the statistical significance of the association index (Ai) and parsimony scores (Ps) for the selected discrete traits using the Bayesian Tip-Significance Testing (BaTS) software version 2.0^86^ to evaluate their contribution to the structure of the inferred posterior MCC trees.

## Supplementary Information

Supplementary Information.

## Data Availability

All the sequences, included in the ML tree analysis and phylodynamic analysis, are available in the GenBank. Accession numbers of sequences included in the phylodynamic analysis are listed in Supplementary Table 1. Alignment, BEAST XML (For geographic and host evolutionary analyses), MCC tree files can be found in the ‘github’: https://github.com/maalkhamis/BTV-phylodynamics.
