# Supplementary material for: Global emergence and evolutionary dynamics of bluetongue virus
Source: Sci Rep. 2020 Dec 10;10:21677. doi: 10.1038/s41598-020-78673-9 (PMC7729867; doi:10.1038/s41598-020-78673-9)
Supplement: Supplementary file 1 — Supplementary Information. [file 41598_2020_78673_MOESM1_ESM.docx]

# Global Emergence and Evolutionary Dynamics of Bluetongue Virus

Moh A. Alkhamis^1^*, Cecilia Aguilar-Vega^2^, Nicholas M. Fountain-Jones^3,4^, Kai Lin^3^, Andres M. Perez3, José M. Sánchez-Vizcaíno^2^

^1^ Faculty of Public Heath, Health Sciences Centre, Kuwait University, Kuwait

^2^VISAVET Health Surveillance Centre and Animal Health Department, Veterinary School, Complutense University of Madrid, Madrid, Spain

^3^ Department of Veterinary Population Medicine, College of Veterinary Medicine, University of Minnesota, St. Paul, United States of America

^4^Department of Maths and Physics, University of Tasmania, Australia

*Corresponding author:

E-mail: [m.alkhamis@hsc.edu.kw](mailto:m.alkhamis@hsc.edu.kw) / [malkahmi@umn.edu](mailto:malkahmi@umn.edu)

Address: Faculty of Public Heath, Health Sciences Center, Kuwait University, Kuwait, Street 109, Jabriya Campus, P.O. Box 24923, 13110  Safat  Kuwait. Tel: + (965) 25347923.

**Appendix**

Supplementary Figure 1 – page 2

Supplementary Table 1 – page 3-12

Supplementary Table 2 – page 13-21

Supplementary Table 3 – page 22-23

Supplementary Table 4 – page 22-25

Supplementary Table 5 – page 26

Supplementary Table 6 – page 27

Supplementary Table 7 – page 22-25


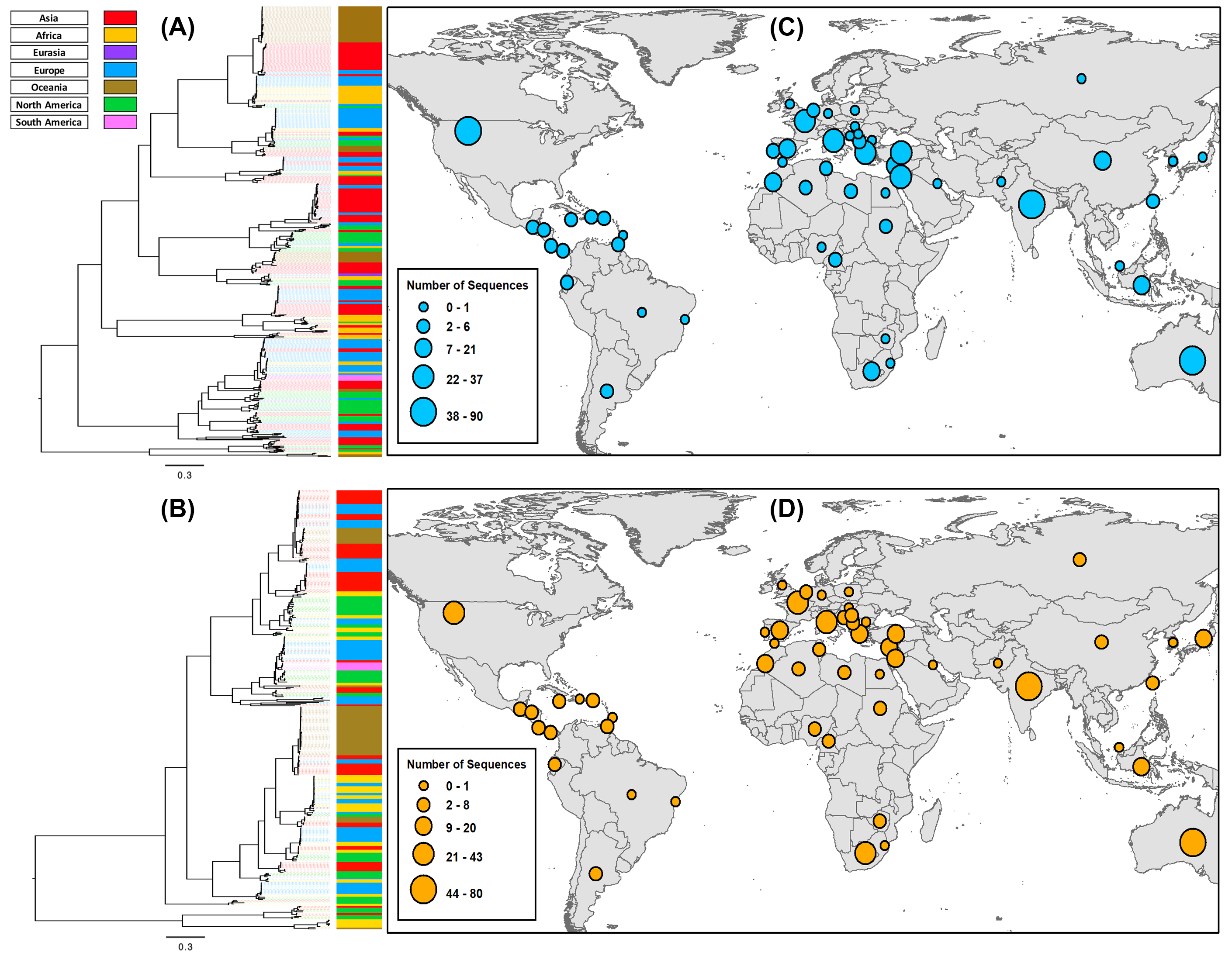


**Fig S1. Maximum likelihood phylogeny and global geographical distribution of segments 10 (n = 558) and 6 (n = 487) sequences of Bluetongue virus between 1937 and 2016.** (A-B) The trees of both segments were constructed using the GTR+Γ substitution model. Support given at nodes based on through bootstrap search using 10 runs, with 100 ML replicates in each run, implemented in RAxML version 8. Scale bar indicate substitution rate per site. (C-D) Circles’ size is proportional to the number of isolates. (C) Blue circles indicate segment 10. (D) Yellow circles indicate segment 6. Maps were generated using ArcGIS version 10.5 (https://www.esri.com).

**Table S1. Summary profile of segment 10 gene sequences used for BTV phylodynamics analyses (n=389).**

| ID | Accession | Isolate name | Serotype | Country | Host | Year |
| --- | --- | --- | --- | --- | --- | --- |
| 1 | KP822003 | CYP1958/01 | 3 | Cyprus | Unknown | 1958 |
| 2 | KP822005 | CYP1969/01 | 4 | Cyprus | Cattle | 1969 |
| 3 | KP822012 | EGY1977/01 | 4 | Egypt | Unknown | 1977 |
| 4 | JQ086260 | CSIRO19 | 20 | Australia | Culicoides | 1977 |
| 5 | JN881994 | CSIRO156 | 1 | Australia | Cattle | 1979 |
| 6 | JQ086270 | DPP86 | 21 | Australia | Cattle | 1979 |
| 7 | AF044386 | 11O79X | 11 | USA | Sheep | 1979 |
| 8 | AF044706 | 17O79Y | 17 | USA | Sheep | 1979 |
| 9 | AF044379 | 10B80Z | 10 | USA | Cattle | 1980 |
| 10 | AF044380 | 10O80Z | 10 | USA | Sheep | 1980 |
| 11 | AF044702 | 11B80Z | 11 | USA | Cattle | 1980 |
| 12 | AF044711 | 13B80Z | 13 | USA | Cattle | 1980 |
| 13 | KM099659 | DPP0065 | 1 | Australia | Cattle | 1981 |
| 14 | MF384939 | E90 | 21 | Australia | Cattle | 1981 |
| 15 | AF044381 | 10B81U | 10 | USA | Cattle | 1981 |
| 16 | AF044382 | 10B81X | 10 | USA | Cattle | 1981 |
| 17 | AF044703 | 11C81Z | 11 | USA | Goat | 1981 |
| 18 | AF044704 | 11O81X | 11 | USA | Sheep | 1981 |
| 19 | AF044707 | 17B81Y | 17 | USA | Cattle | 1981 |
| 20 | AF044712 | 13B81K | 13 | USA | Cattle | 1981 |
| 21 | KP822059 | CAR1982/04 | 14 | Cameroon | Unknown | 1982 |
| 22 | JQ713563 | IND1982/01 | 2 | India | Unknown | 1982 |
| 23 | JQ086280 | DPP90 | 23 | Australia | Cattle | 1982 |
| 24 | JQ086230 | DPP192 | 15 | Australia | Cattle | 1982 |
| 25 | KC853058 | NIG1982/10 | 16 | Nigeria | Unknown | 1982 |
| 26 | KP821963 | CAR1982/01 | 1 | Cameroon | Sheep | 1982 |
| 27 | KP822036 | SUD1983/01 | 4 | Sudan | Cattle | 1983 |
| 28 | KP822001 | SUD1983/05 | 2 | Sudan | Culicoides | 1983 |
| 29 | KP696516 | IND1985/01 | 1 | India | Unknown | 1985 |
| 30 | JQ086310 | DP0837 | 9 | Australia | Cattle | 1985 |
| 31 | KM099660 | DPP1000 | 1 | Australia | Cattle | 1986 |
| 32 | JQ086290 | DPP973 | 3 | Australia | Cattle | 1986 |
| 33 | KP821987 | SUD1987/01 | 1 | Sudan | Unknown | 1987 |
| 34 | JQ086240 | DPP965 | 16 | Australia | Cattle | 1987 |
| 35 | KM099661 | DPP1315 | 1 | Australia | Cattle | 1988 |
| 36 | JQ771831 | IND1988/02 | 23 | India | Sheep | 1988 |
| 37 | AY426595 | BT12-JAM2016 | 12 | Jamaica | Unknown | 1988 |
| 38 | AY426599 | BT3-2058 | 3 | Costa Rica | Unknown | 1988 |
| 39 | KY091903 | BTV3 BAR1988/502034 | 3 | Barbados | Unknown | 1988 |
| 40 | KY091906 | BTV3 JAM1988/502031 | 3 | Jamaica | Unknown | 1988 |
| 41 | KM099662 | DPP1523 | 1 | Australia | Cattle | 1989 |
| 42 | MF384968 | RIVS-66 | 21 | Indonesia | Cattle | 1989 |
| 43 | MF384941 | E051 | 1 | Australia | Cattle | 1989 |
| 44 | MF384940 | E050 | 1 | Australia | Cattle | 1989 |
| 45 | AY426598 | BT1-2172 | 1 | Honduras | Unknown | 1989 |
| 46 | KY091907 | BTV3 PAN1989/502151 | 3 | Panama | Unknown | 1989 |
| 47 | KY091921 | BTV9 PAN1989/502130 | 9 | Panama | Unknown | 1989 |
| 48 | KY091925 | BTV14 TOBG1989/502091 | 14 | Trinidad and Tobago | Unknown | 1989 |
| 49 | KY091927 | BTV22 TRIN1989/502165 | 22 | Trinidad and Tobago | Unknown | 1989 |
| 50 | AF044710 | 13B89Z | 13 | USA | Cattle | 1989 |
| 51 | KF986491 | 185 | 2 | Panama | Cattle | 1989 |
| 52 | AF529059 | RIVS 60 | 23 | Indonesia | Cattle | 1990 |
| 53 | AF529050 | RIVS 53 | 3 | Indonesia | Cattle | 1990 |
| 54 | AF529049 | RIVS 46 | 1 | Indonesia | Cattle | 1990 |
| 55 | MF384996 | RIVS-60 | 21 | Indonesia | Cattle | 1990 |
| 56 | MF384992 | RIVS-160 | 16 | Indonesia | Cattle | 1990 |
| 57 | MF384953 | RIVS-53 | 3 | Indonesia | Cattle | 1990 |
| 58 | MF384952 | RIVS-63 | 21 | Indonesia | Cattle | 1990 |
| 59 | AY426596 | BT17-283 | 17 | Puerto Rico | Unknown | 1990 |
| 60 | AY426597 | BT17-298 | 17 | Puerto Rico | Unknown | 1990 |
| 61 | AY426600 | BT3-2230 | 3 | Guatemala | Unknown | 1990 |
| 62 | AY426602 | BT4-2227 | 4 | Dominican Republic | Unknown | 1990 |
| 63 | AY426603 | BT6-2187 | 6 | Honduras | Unknown | 1990 |
| 64 | AY426604 | BT8-2215 | 8 | Dominican Republic | Unknown | 1990 |
| 65 | KY091901 | BTV1 SAL1990/502270 | 1 | Salvador | Unknown | 1990 |
| 66 | KY091904 | BTV3 HON1990/502285 | 3 | Honduras | Unknown | 1990 |
| 67 | KY091926 | BTV18 GUA1990/502229 | 18 | Guatemala | Unknown | 1990 |
| 68 | AF044384 | 10B90Z | 10 | USA | Cattle | 1990 |
| 69 | AF044385 | 10O90H | 10 | USA | Sheep | 1990 |
| 70 | AF044708 | 17B90Z | 17 | USA | Cattle | 1990 |
| 71 | AF044709 | 17O90Y | 17 | USA | Sheep | 1990 |
| 72 | AY028210 | 10O90z | 10 | USA | Sheep | 1990 |
| 73 | AF529051 | RIVS 39 | 23 | Indonesia | Cattle | 1991 |
| 74 | MF384995 | RIVS-138 | 21 | Indonesia | Cattle | 1991 |
| 75 | MF384993 | RIVS-113 | 21 | Indonesia | Cattle | 1991 |
| 76 | MF384969 | RIVS-74 | 9 | Indonesia | Cattle | 1991 |
| 77 | KY091920 | BTV9 HON1991/502325 | 9 | Honduras | Unknown | 1991 |
| 78 | JQ282777 | IND1992/01 | 1 | India | Sheep | 1992 |
| 79 | KP696534 | IND1992/02 | 1 | India | Sheep | 1992 |
| 80 | KY091922 | BTV10 CSTRCA1992/502344 | 10 | Costa Rica | Unknown | 1992 |
| 81 | KP696591 | BTV-2/IND1993/01 | 2 | India | Sheep | 1993 |
| 82 | KP339163 | BTV02IND1993 | 2 | India | Sheep | 1993 |
| 83 | KP268783 | IND1994/01 | 2 | India | Unknown | 1994 |
| 84 | EF540909 | 10US94-1 | 17 | USA | Deer | 1994 |
| 85 | KX424875 | EMBOSS_001 | 2 | India | Sheep | 1995 |
| 86 | JN671915 | BN96/16 | 16 | China | Sheep | 1996 |
| 87 | JX560422 | YTS-4 | 4 | China | Cattle | 1997 |
| 88 | JN848768 | SZ97/1 | 1 | China | Sheep | 1997 |
| 89 | KP822040 | RSA1998/01 | 8 | South Africa | Goat | 1998 |
| 90 | EF540911 | 10US98-3 | 10 | USA | Unknown | 1998 |
| 91 | MF384999 | G087 | 21 | Australia | Cattle | 1998 |
| 92 | MF384951 | G089 | 21 | Australia | Cattle | 1998 |
| 93 | MF384950 | G088 | 21 | Australia | Cattle | 1998 |
| 94 | AY677629 | GR199/98RS | 9 | Greece | Sheep | 1998 |
| 95 | JQ424791 | GP/CHT/1998 | Unknown | India | Sheep | 1998 |
| 96 | KP822056 | TUR1998/02 | 9 | Turkey | Sheep | 1998 |
| 97 | KP822045 | GRE1998/01 | 9 | Greece | Sheep | 1998 |
| 98 | KP696543 | IND1999/01 | 1 | India | Sheep | 1999 |
| 99 | EF554854 | BTV_TR22 | 9 | Turkey | Sheep | 1999 |
| 100 | EF554852 | BTV_TR20 | 9 | Turkey | Sheep | 1999 |
| 101 | EF554851 | BTV_TR19 | 16 | Turkey | Sheep | 1999 |
| 102 | EF554850 | BTV_TR18 | 9 | Turkey | Sheep | 1999 |
| 103 | EF554846 | BTV_TR9 | 9 | Turkey | Sheep | 1999 |
| 104 | EF554845 | BTV_TR8 | 9 | Turkey | Unknown | 1999 |
| 105 | EF554843 | BTV_TR6 | 9 | Turkey | Sheep | 1999 |
| 106 | EF554841 | BTV_TR4 | 9 | Turkey | Sheep | 1999 |
| 107 | EF554840 | BTV_TR3 | 9 | Turkey | Sheep | 1999 |
| 108 | EF554839 | BTV_TR2 | 9 | Turkey | Sheep | 1999 |
| 109 | MF384997 | 299 | 1 | Australia | Cattle | 1999 |
| 110 | AY691691 | GR280/99RoS | 4 | Greece | Sheep | 1999 |
| 111 | AY677627 | GR610/99EvS | 4 | Greece | Sheep | 1999 |
| 112 | AY677620 | GR378/99CB | 4 | Greece | Cattle | 1999 |
| 113 | AY449652 | GR308/99RS | 16 | Greece | Sheep | 1999 |
| 114 | EF540912 | 13US99-4 | 13 | USA | Deer | 1999 |
| 115 | AY120938 | GA05 | 8 | South Africa | Unknown | 1999 |
| 116 | AF512905 | G712/1999 | 4 | South Africa | Unknown | 1999 |
| 117 | AF512906 | G717/1999 | 3 | South Africa | Unknown | 1999 |
| 118 | AF512907 | P524/1999 | 4 | South Africa | Unknown | 1999 |
| 119 | AF512912 | S1/1999 | 1 | India | Unknown | 1999 |
| 120 | AF512914 | B1/1999 | 18 | India | Unknown | 1999 |
| 121 | AF512920 | Ramsen/1999 | 2 | South Africa | Unknown | 1999 |
| 122 | AF512921 | G704/1999 | 11 | South Africa | Unknown | 1999 |
| 123 | KM099668 | DPP4690 | 1 | Australia | Cattle | 1999 |
| 124 | JQ080436 | NA | Unknown | India | Sheep | 1999 |
| 125 | KP822046 | GRE1999/06 | 9 | Greece | Sheep | 1999 |
| 126 | KP822044 | BUL1999/01 | 9 | Bulgaria | Unknown | 1999 |
| 127 | KP822018 | GRE1999/26 | 4 | Greece | Culicoides | 1999 |
| 128 | KP822047 | GRE1999/31 | 9 | Greece | Cattle | 1999 |
| 129 | JX024944 | 4/ARG/2001/99 | 4 | Argentina | Cattle | 1999 |
| 130 | JX024949 | 4/ARG/2001/102 | 4 | Argentina | Cattle | 1999 |
| 131 | JX024954 | 4/ARG/2001/829 | 4 | Argentina | Cattle | 1999 |
| 132 | KP822002 | TUN2000/01 | 2 | Tunisia | Unknown | 2000 |
| 133 | KP822057 | TUR2000/03 | 9 | Turkey | Unknown | 2000 |
| 134 | KP821959 | TUR2000/01 | 16 | Turkey | Unknown | 2000 |
| 135 | JN255921 | BTV-9IT(H) | 9 | Italy | Sheep | 2000 |
| 136 | JN255881 | BTV-2IT(H) | 2 | Italy | Sheep | 2000 |
| 137 | AY857505 | 2000 | 4 | Greece | Goat | 2000 |
| 138 | EF554853 | BTV_TR21 | 9 | Turkey | Sheep | 2000 |
| 139 | EF554849 | BTV_TR12 | 9 | Turkey | Unknown | 2000 |
| 140 | EF554848 | BTV_TR11 | 9 | Turkey | Sheep | 2000 |
| 141 | EF554847 | BTV_TR10 | 9 | Turkey | Sheep | 2000 |
| 142 | EF554842 | BTV_TR5 | 9 | Turkey | Unknown | 2000 |
| 143 | MF384973 | 705 | 1 | Australia | Cattle | 2000 |
| 144 | MF384963 | G092 | 21 | Australia | Cattle | 2000 |
| 145 | MF384959 | G093 | 1 | Australia | Cattle | 2000 |
| 146 | EF540914 | 17US00-6 | 17 | USA | Deer | 2000 |
| 147 | EF540913 | 17US00-5 | 17 | USA | Unknown | 2000 |
| 148 | AY823220 | NA | 2 | Italy | Sheep | 2000 |
| 149 | KX650180 | BTV-2/IND2000 | 2 | India | Sheep | 2000 |
| 150 | AF481092 | Corsican bluetongue (2000) | 2 | France | Unknown | 2000 |
| 151 | KM053277 | BTV-2IT2000 | 2 | Italy | Sheep | 2000 |
| 152 | KP822058 | TUR2001/02 | 9 | Turkey | Sheep | 2001 |
| 153 | KP821992 | ISR2001/17 | 2 | Israel | Sheep | 2001 |
| 154 | KP821956 | ISR2001/18 | 16 | Israel | Sheep | 2001 |
| 155 | KP696551 | IND2001/01 | 1 | India | Culicoides | 2001 |
| 156 | KP821989 | FRA2001/01 | 2 | France | Sheep | 2001 |
| 157 | EF554858 | BTV_TR26 | 9 | Turkey | Sheep | 2001 |
| 158 | EF554857 | BTV_TR25 | 9 | Turkey | Sheep | 2001 |
| 159 | EF554856 | BTV_TR24 | 9 | Turkey | Sheep | 2001 |
| 160 | EF554855 | BTV_TR23 | Unknown | Turkey | Sheep | 2001 |
| 161 | MF384974 | 837 | 1 | Australia | Cattle | 2001 |
| 162 | AY677628 | GR1472/01LS | 1 | Greece | Sheep | 2001 |
| 163 | AY823221 | 81640_01 | 2 | Italy | Sheep | 2001 |
| 164 | KY091910 | BTV3 USA2001/FL 138555-30 | 3 | USA | Deer | 2001 |
| 165 | AY775158 | 320226 | 4 | Israel | Sheep | 2001 |
| 166 | AY775157 | 322222 | 4 | Israel | Sheep | 2001 |
| 167 | AF481093 | Corsican bluetongue (2001) | 2 | France | Sheep | 2001 |
| 168 | KP822050 | KOS2001/03 | 9 | Kosovo | Sheep | 2001 |
| 169 | KP822049 | KOS2001/01 | 9 | Kosovo | Sheep | 2001 |
| 170 | KP821991 | FRA2001/06 | 2 | France | Sheep | 2001 |
| 171 | KP821972 | GRE2001/10 | 1 | Greece | Sheep | 2001 |
| 172 | KP821998 | SAD2001/03 | 2 | Italy | Sheep | 2001 |
| 173 | KP821999 | SAD2002/02 | 2 | Italy | Sheep | 2002 |
| 174 | KM099669 | DPP5775 | 1 | Australia | Cattle | 2002 |
| 175 | KM099670 | DPP5844 | 1 | Australia | Cattle | 2002 |
| 176 | KF387530 | ITL2002 | 16 | Italy | Cattle | 2002 |
| 177 | KP821994 | ITL2002/07 | 2 | Italy | Sheep | 2002 |
| 178 | EF540917 | 10US02-9 | 10 | USA | Deer | 2002 |
| 179 | EF540916 | 10US02-8 | 10 | USA | Deer | 2002 |
| 180 | MF384987 | 999 | 21 | Australia | Cattle | 2002 |
| 181 | MF384975 | 973 | 1 | Australia | Cattle | 2002 |
| 182 | KY091911 | BTV3 USA2002/FL 202795 | 3 | USA | Deer | 2002 |
| 183 | KY091912 | BTV3 USA2002/FL 220082-16 | 3 | USA | Cattle | 2002 |
| 184 | AY775159 | 2002 | 4 | Greece | Unknown | 2002 |
| 185 | AY485668 | la Reunion | 3 | France | Cattle | 2002 |
| 186 | KP822043 | BOS2002/02 | 9 | Bosnia and Herzegovina | Sheep | 2002 |
| 187 | KP822048 | ITL2003/01 | 9 | Italy | Sheep | 2003 |
| 188 | KP822015 | FRA2003-200 | 4 | France | Unknown | 2003 |
| 189 | KP696581 | BTV-1/IND2003/10 | 1 | India | Sheep | 2003 |
| 190 | KP696611 | BTV-2/IND2003/03 | 2 | India | Sheep | 2003 |
| 191 | KP696601 | BTV-2/IND2003/01 | 2 | India | Sheep | 2003 |
| 192 | KP696571 | BTV-1/IND2003/05 | 1 | India | Sheep | 2003 |
| 193 | KP696561 | IND2003/04 | 1 | India | Sheep | 2003 |
| 194 | JQ771822 | IND2003/08 | 3 | India | Unknown | 2003 |
| 195 | JN255891 | BTV-4IT(L) | 4 | Italy | Sheep | 2003 |
| 196 | JN255901 | BTV-4IT(H) | 4 | Italy | Sheep | 2003 |
| 197 | AY857503 | Corsica | 4 | France | Unknown | 2003 |
| 198 | EF540929 | 10US03-25 | 10 | USA | Sheep | 2003 |
| 199 | EF540918 | 17US03-10 | 17 | USA | Deer | 2003 |
| 200 | MF384976 | 1121 | 1 | Australia | Cattle | 2003 |
| 201 | MF384960 | G095 | 1 | Australia | Cattle | 2003 |
| 202 | EF540928 | 17US03-24 | 17 | USA | Deer | 2003 |
| 203 | EF540927 | 13US03-23 | 13 | USA | Sheep | 2003 |
| 204 | EF540926 | 17US03-21 | 17 | USA | Cattle | 2003 |
| 205 | EF540925 | 17US03-20 | 17 | USA | Cattle | 2003 |
| 206 | EF174159 | BTV12/PT/2003 | 12 | Taiwan | Cattle | 2003 |
| 207 | KY091913 | BTV3 USA2003/FL 280559-9 | 3 | USA | Cattle | 2003 |
| 208 | AY493685 | KM | 2 | Taiwan | Goat | 2003 |
| 209 | KP339193 | BTV09IND2003-M11 | 9 | India | Sheep | 2003 |
| 210 | KP339183 | BTV09IND2003-M10 | 9 | India | Sheep | 2003 |
| 211 | KX164118 | USA2003/FL 279313 | 14 | USA | Sheep | 2003 |
| 212 | KX164138 | USA2003/FL 280559-3 | 19 | USA | Cattle | 2003 |
| 213 | KX164068 | USA2003/FL 280559-7 | 5 | USA | Cattle | 2003 |
| 214 | KF986511 | USA2003 | 2 | USA | Cattle | 2003 |
| 215 | AB504736 | ON-2/C/03 | 9 | Japan | Culicoides | 2003 |
| 216 | KM099671 | DPP6112 | 1 | Australia | Cattle | 2004 |
| 217 | KP821953 | COR2004/01 | 16 | France | Sheep | 2004 |
| 218 | JQ740780 | IND2004/01 | 10 | India | Unknown | 2004 |
| 219 | KP696699 | BTV-23/IND2004/09 | 23 | India | Unknown | 2004 |
| 220 | KP696689 | BTV-23/IND2004/08 | 23 | India | Unknown | 2004 |
| 221 | KP696621 | BTV-9/IND2004/02 | 9 | India | Unknown | 2004 |
| 222 | EF540933 | 17US04-30 | 17 | USA | Deer | 2004 |
| 223 | EF540932 | 17US04-29 | 17 | USA | Deer | 2004 |
| 224 | EF434180 | BTV4/22045/PT04 | 4 | Portugal | Sheep | 2004 |
| 225 | KP822026 | MOR2004/02 | 4 | Morocco | Sheep | 2004 |
| 226 | KM099672 | DPP6504 | 1 | Australia | Cattle | 2005 |
| 227 | KP696660 | BTV-9/IND2005/03 | 9 | India | Sheep | 2005 |
| 228 | KP822000 | SPA2005/01 | 2 | Spain | Cattle | 2005 |
| 229 | JQ424789 | M11/MBN/2005 | 9 | India | Sheep | 2005 |
| 230 | JQ424788 | M10/MBN/2005 | Unknown | India | Sheep | 2005 |
| 231 | EF434179 | BTV2/26629/PT05 | 2 | Portugal | Cattle | 2005 |
| 232 | KP339243 | KMN07/05 | 21 | India | Sheep | 2005 |
| 233 | KX164148 | USA2005/FL 402286 | 22 | USA | Sheep | 2005 |
| 234 | KJ872782 | Ardennes | 8 | France | Sheep | 2006 |
| 235 | KJ736010 | SAD2006 | 1 | Italy | Sheep | 2006 |
| 236 | EF540935 | 10FMI06-33 | 10 | Martinique | Cattle | 2006 |
| 237 | EF540936 | 17FMI06-34 | 17 | Martinique | Cattle | 2006 |
| 238 | EF540934 | 2FMI06-32 | 2 | Martinique | Cattle | 2006 |
| 239 | KY091914 | BTV3 USA2006/MS 473906-7 | 3 | USA | Deer | 2006 |
| 240 | JX680456 | NET2006/04 | 8 | Netherlands | Cattle | 2006 |
| 241 | KP821961 | ALG2006/04 | 1 | Algeria | Sheep | 2006 |
| 242 | KP821974 | MOR2006/06 | 1 | Morocco | Sheep | 2006 |
| 243 | JF443165 | BBF | 9 | India | Sheep | 2006 |
| 244 | GQ506545 | USA2006/01 | 6 | USA | Cattle | 2006 |
| 245 | KP822063 | ISR2006/11 | 15 | Israel | Sheep | 2006 |
| 246 | KP822024 | ISR2006/12 | 4 | Israel | Sheep | 2006 |
| 247 | KF986498 | USA2006 | 2 | USA | Cattle | 2006 |
| 248 | KP821984 | SPA2007/05 | 1 | Spain | Cattle | 2007 |
| 249 | KP821983 | SPA2007/04 | 1 | Spain | Cattle | 2007 |
| 250 | JQ086300 | v6963 | 7 | Australia | Cattle | 2007 |
| 251 | KP821978 | MOR2007/04 | 1 | Morocco | Sheep | 2007 |
| 252 | MF384978 | 1923 | 1 | Australia | Cattle | 2007 |
| 253 | KP821973 | LIB2007/06 | 1 | Libya | Sheep | 2007 |
| 254 | KP822042 | UKG2007/06 | 8 | United Kingdom | Cattle | 2007 |
| 255 | KP821988 | TUN2007/01 | 1 | Tunisia | Sheep | 2007 |
| 256 | KP821969 | GIB2007/06 | 1 | Gibraltar | Sheep | 2007 |
| 257 | KX164158 | USA2007/FL 520518 | 24 | USA | Deer | 2007 |
| 258 | JX003696 | Ind-R1-2007 | 9 | India | Sheep | 2007 |
| 259 | FJ437561 | btv1/07-01 | 1 | France | Sheep | 2007 |
| 260 | KM099673 | DPP7137 | 1 | Australia | Cattle | 2008 |
| 261 | KP822038 | GRE2008/01 | 8 | Greece | Sheep | 2008 |
| 262 | JQ904064 | Sheep/08/Ind/ABT/Hisar | 2 | India | Sheep | 2008 |
| 263 | KF664132 | NRT37/ABT/HSR | 1 | India | Sheep | 2008 |
| 264 | KF664112 | K31-08/ABT/HSR | 16 | India | Sheep | 2008 |
| 265 | JQ086250 | v7291 | 2 | Australia | Cattle | 2008 |
| 266 | KX164088 | USA2008/TX 576181 | 12 | USA | Deer | 2008 |
| 267 | KX164038 | USA2008/AR 566195 | 3 | USA | Deer | 2008 |
| 268 | KU234266 | MKD20/08/Ind | 1 | India | Goat | 2008 |
| 269 | KP196612 | BT 57/08 | 29 | South Africa | Sheep | 2008 |
| 270 | KM053267 | BTV-8IT2008 | 8 | Italy | Sheep | 2008 |
| 271 | KP822039 | NET2008/03 | 8 | Netherlands | Cattle | 2008 |
| 272 | KP821965 | FRA2008/22 | 1 | France | Sheep | 2008 |
| 273 | KP821945 | FRA2008/27 | 8 | France | Cattle | 2008 |
| 274 | JX861497 | FRA2008/24 | 1 | France | Cattle | 2008 |
| 275 | GQ506481 | NET2008/05 | 6 | Netherlands | Cattle | 2008 |
| 276 | KP822052 | LIB2008/03 | 9 | Libya | Sheep | 2008 |
| 277 | KP822051 | LIB2008/08 | 9 | Libya | Sheep | 2008 |
| 278 | GQ506487 | NET2008/06 | 6 | Netherlands | Cattle | 2008 |
| 279 | JX399157 | K23/08 | 1 | India | Sheep | 2008 |
| 280 | KP821955 | GRE2008/10 | 16 | Greece | Sheep | 2008 |
| 281 | KP822025 | ISR2008/02 | 4 | Israel | Cattle | 2008 |
| 282 | KP821957 | ISR2008/03 | 16 | Israel | Goat | 2008 |
| 283 | KX164078 | USA2008/FL 576307 | 9 | USA | Deer | 2008 |
| 284 | KP821962 | ALG2008/10 | 1 | Algeria | Sheep | 2008 |
| 285 | KP822037 | ISR2008/13 | 8 | Israel | Cattle | 2008 |
| 286 | JX889179 | BTV24/2305/08 | 24 | Israel | Sheep | 2008 |
| 287 | GU954427 | B09 | Unknown | USA | Cattle | 2009 |
| 288 | GU954426 | B09 | Unknown | USA | Cattle | 2009 |
| 289 | GU954425 | 11B09 | 11 | USA | Cattle | 2009 |
| 290 | MF384944 | E047 | 1 | Australia | Cattle | 2009 |
| 291 | KY091916 | BTV3 USA2009/MS 025763-326 | 3 | USA | Deer | 2009 |
| 292 | JX024959 | 4/ARG/2009 | 4 | Argentina | Cattle | 2009 |
| 293 | KP821981 | MOR2009/06 | 1 | Morocco | Sheep | 2009 |
| 294 | KP821980 | MOR2009/04 | 1 | Morocco | Sheep | 2009 |
| 295 | KP822030 | MOR2009/10 | 4 | Morocco | Sheep | 2009 |
| 296 | KP822029 | MOR2009/09 | 4 | Morocco | Sheep | 2009 |
| 297 | KP822028 | MOR2009/08 | 4 | Morocco | Sheep | 2009 |
| 298 | KP822027 | MOR2009/07 | 4 | Morocco | Sheep | 2009 |
| 299 | KM099674 | DPP8086 | 1 | Australia | Cattle | 2010 |
| 300 | KF584206 | BTV8/1206/10 | 8 | Israel | Cattle | 2010 |
| 301 | JQ972860 | BTV-11_DE | 11 | Germany | Cattle | 2010 |
| 302 | JQ972870 | BTV-11_MQ | 11 | Martinique | Cattle | 2010 |
| 303 | JQ822257 | USA2010 | 2 | USA | Cattle | 2010 |
| 304 | JQ924829 | IND/Goat/2010/16/HSR | 16 | India | Goat | 2010 |
| 305 | MF384990 | v8184 | 21 | Australia | Cattle | 2010 |
| 306 | MF384985 | 2010-90 | 2 | Australia | Cattle | 2010 |
| 307 | MF384983 | 2010-21 | 2 | Australia | Cattle | 2010 |
| 308 | MF384967 | G100 | 21 | Australia | Cattle | 2010 |
| 309 | JQ240330 | Cooktown | 2 | Australia | Cattle | 2010 |
| 310 | KM099675 | DPP8304 | 1 | Australia | Cattle | 2010 |
| 311 | KP339233 | BTV16IND2010-VC07 | 16 | India | Sheep | 2010 |
| 312 | KP339173 | BTV02IND2010-KRM08 | 2 | India | Sheep | 2010 |
| 313 | KP339153 | BTV01IND2010-VC12 | 1 | India | Sheep | 2010 |
| 314 | KP339143 | BTV01IND2010-KRM07 | 1 | India | Sheep | 2010 |
| 315 | KP822035 | SPA2010/01 | 4 | Spain | Cattle | 2010 |
| 316 | JX024964 | 4/ARG/2010 | 4 | Argentina | Cattle | 2010 |
| 317 | JX889173 | BTV24/2214/1/10 | 24 | Israel | Sheep | 2010 |
| 318 | KX164028 | USA2010/FL 10-044273 | 1 | USA | Sheep | 2010 |
| 319 | KP339213 | BTV16IND2010-AP06 | 16 | India | Sheep | 2010 |
| 320 | KP339203 | BTV16IND2010-AP04 | 16 | India | Sheep | 2010 |
| 321 | KF460445 | Krishna | 2 | India | Sheep | 2010 |
| 322 | JX007931 | IND2010/Cattle/16 | 16 | India | Cattle | 2010 |
| 323 | JX889191 | BTV24/2755/1/10 | 24 | Israel | Sheep | 2010 |
| 324 | KF460444 | Guntur | 2 | India | Sheep | 2010 |
| 325 | KJ577123 | SAD2010 | 1 | Italy | Sheep | 2010 |
| 326 | KP821952 | CYP2010/03 | 16 | Cyprus | Cattle | 2010 |
| 327 | JX889198 | BTV24/2944/1/10 | 24 | Israel | Sheep | 2010 |
| 328 | JX889203 | BTV24/3027/1/10 | 24 | Israel | Sheep | 2010 |
| 329 | JX889208 | BTV24/3027/6/10 | 24 | Israel | Sheep | 2010 |
| 330 | JX889214 | BTV24/3258/1/10 | 24 | Israel | Cattle | 2010 |
| 331 | KJ577113 | TUN2011 | 1 | Tunisia | Sheep | 2011 |
| 332 | KF664142 | G53/ABT/HSR | 16 | India | Goat | 2011 |
| 333 | KM580421 | USA2011/TX 11-56803-3 | 11 | USA | Deer | 2011 |
| 334 | KM580429 | USA2011/TX 11-56803-5 | 11 | USA | Deer | 2011 |
| 335 | KP339223 | BTV16IND2011-NR82 | 16 | India | Sheep | 2011 |
| 336 | KP822061 | RUS2011/01 | 14 | Russia | Cattle | 2011 |
| 337 | KY049852 | BTV-1/11-01 (4074) | 1 | Guyane | Cattle | 2011 |
| 338 | KC662621 | INDAPADBNMO1/11 | 12 | India | Sheep | 2011 |
| 339 | KM580440 | USA2011/TX 11-56803-9 | 11 | USA | Deer | 2011 |
| 340 | KP822008 | CYP2011/02 | 4 | Cyprus | Sheep | 2011 |
| 341 | KP822007 | CYP2011/01 | 4 | Cyprus | Sheep | 2011 |
| 342 | KM580455 | USA2011/TX 128184-11 | 11 | USA | Deer | 2011 |
| 343 | KF584205 | BTV5/1405/11 | 5 | Israel | Cattle | 2012 |
| 344 | KC896853 | BTV4 SAD2012 | 4 | Italy | Sheep | 2012 |
| 345 | MF384998 | 2012-31 | 21 | Australia | Cattle | 2012 |
| 346 | MF384991 | 2012-26 | 1 | Australia | Cattle | 2012 |
| 347 | MF384989 | 2012-44 | 21 | Australia | Cattle | 2012 |
| 348 | MF384988 | 2012-33 | 21 | Australia | Cattle | 2012 |
| 349 | MF384986 | 2012-38 | 15 | Australia | Cattle | 2012 |
| 350 | MF384982 | 2012-29 | 1 | Australia | Cattle | 2012 |
| 351 | MF384981 | 2012-12 | 1 | Australia | Cattle | 2012 |
| 352 | KP822062 | SPA2012/01 | 14 | Spain | Cattle | 2012 |
| 353 | KX164048 | USA2012/SD 12-035694 | 3 | USA | Deer | 2012 |
| 354 | KF584169 | BTV2/1841/3/12 | 2 | Israel | Cattle | 2012 |
| 355 | KF584189 | BTV4/1855/2/12 | 4 | Israel | Sheep | 2012 |
| 356 | KX164098 | USA2012/LA 12-046093 | 12 | USA | Deer | 2012 |
| 357 | KY513439 | BTV2/ISR-1894/12 | 2 | Israel | Sheep | 2012 |
| 358 | KF584175 | BTV2/1981/12 | 2 | Israel | Unknown | 2012 |
| 359 | KJ577103 | SAD2012 | 1 | Italy | Sheep | 2012 |
| 360 | KF584209 | BTV16/1974/4/12 | 16 | Israel | Cattle | 2012 |
| 361 | KP822023 | GRE2012/03 | 4 | Greece | Sheep | 2012 |
| 362 | KF584178 | BTV2/2069/5/12 | 2 | Israel | Sheep | 2012 |
| 363 | KF584182 | BTV2/2091/2/12 | 2 | Israel | Unknown | 2012 |
| 364 | KF584199 | BTV4/2091/1/12 | 4 | Israel | Cattle | 2012 |
| 365 | KF584184 | BTV2/2115/4/12 | 2 | Israel | Cattle | 2012 |
| 366 | KY091918 | BTV3 USA2013/FL N13-03980 | 3 | USA | Deer | 2013 |
| 367 | KM580488 | USA2013/WA 13-031503 | 11 | USA | Deer | 2013 |
| 368 | KX164108 | USA2013/CA 13-034210 | 13 | USA | Deer | 2013 |
| 369 | KJ577133 | LAZ2013 | 1 | Italy | Sheep | 2013 |
| 370 | KJ661738 | SIC2013 | 1 | Italy | Sheep | 2013 |
| 371 | KM580474 | USA2013/FL 13-037190 | 11 | USA | Deer | 2013 |
| 372 | LN713679 | 379 | 27 | France | Goat | 2014 |
| 373 | KX599368 | 17/BRA/2014/73 | 17 | Brazil | Sheep | 2014 |
| 374 | KP268823 | BTV4-HUN2014 | 4 | Hungary | Cattle | 2014 |
| 375 | KU761006 | BTV-27/FRA2014/v03 | 27 | France | Goat | 2014 |
| 376 | KR085419 | XJ1407 | Unknown | China | Goat | 2014 |
| 377 | KT002587 | GDST008 | 7 | China | Cattle | 2014 |
| 378 | KX695179 | V196/XJ/2014 | Unknown | China | Goat | 2014 |
| 379 | KX164128 | USA2014/FL 15-008010 | 18 | USA | Deer | 2014 |
| 380 | KX302643 | IND2014/01 | 16 | India | Sheep | 2014 |
| 381 | KY049889 | BTV-18/15-01 (58) | 18 | Ecuador | Cattle | 2015 |
| 382 | KY049880 | BTV-13/15-01 (6) | 13 | Ecuador | Cattle | 2015 |
| 383 | KY049871 | BTV-9/15-01 (7) | 9 | Ecuador | Cattle | 2015 |
| 384 | KY049862 | BTV-1/15.01 (5370) | 1 | Guyane | Cattle | 2015 |
| 385 | KX164058 | USA2015/TX 15-029176 | 3 | USA | Cattle | 2015 |
| 386 | KU569999 | 15-01 | 8 | France | Sheep | 2015 |
| 387 | MG206086 | 5149E | 21 | China | Cattle | 2015 |
| 388 | KX234087 | BTVX ITL2015 34200 | Unknown | Italy | Goat | 2015 |
| 389 | MF615246 | IND2016/118 | 12 | India | Sheep | 2016 |

**Table S2. Summary profile of segment 6 gene sequences used for BTV phylodynamics analyses (n=337).**

| ID | Accession | Isolate name | Serotype | Country | Host | Year |
| --- | --- | --- | --- | --- | --- | --- |
| 1 | KP821520 | CYP1958/01 | 3 | Cyprus | Unknown | 1958 |
| 2 | KP821522 | CYP1969/01 | 4 | Cyprus | Cattle | 1969 |
| 3 | JQ086256 | CSIRO19 | 20 | Australia | Culicoides | 1977 |
| 4 | KP821529 | EGY1977/01 | 4 | Egypt | Unknown | 1977 |
| 5 | JN881990 | CSIRO156 | 1 | Australia | Cattle | 1979 |
| 6 | JQ086266 | DPP86 | 21 | Australia | Cattle | 1979 |
| 7 | KP821533 | GRE1979/01 | 4 | Greece | Sheep | 1979 |
| 8 | KC879620 | Y863 | 1 | China | Sheep | 1979 |
| 9 | KM099591 | DPP0065 | 1 | Australia | Cattle | 1981 |
| 10 | MF384737 | E90 | 21 | Australia | Cattle | 1981 |
| 11 | KP821480 | CAR1982/01 | 1 | Cameroon | Sheep | 1982 |
| 12 | KP821576 | CAR1982/04 | 14 | Cameroon | Unknown | 1982 |
| 13 | JQ086276 | DPP90 | 23 | Australia | Cattle | 1982 |
| 14 | AJ586675 | IND1982/01 | 2 | India | Unknown | 1982 |
| 15 | AJ586694 | NIG1982/10 | 16 | Nigeria | Unknown | 1982 |
| 16 | KP821553 | SUD1983/01 | 4 | Sudan | Cattle | 1983 |
| 17 | KP821518 | SUD1983/05 | 2 | Sudan | Culicoides | 1983 |
| 18 | JQ086306 | DP0837 | 9 | Australia | Cattle | 1985 |
| 19 | KP696512 | IND1985/01 | 1 | India | Unknown | 1985 |
| 20 | KM099592 | DPP1000 | 1 | Australia | Cattle | 1986 |
| 21 | JQ086286 | DPP973 | 3 | Australia | Cattle | 1986 |
| 22 | JQ086236 | DPP965 | 16 | Australia | Cattle | 1987 |
| 23 | KP821504 | SUD1987/01 | 1 | Sudan | Unknown | 1987 |
| 24 | KY092040 | BTV3 BAR1988/502034 | 3 | Barbados | Unknown | 1988 |
| 25 | KY092042 | BTV3 JAM1988/502030 | 3 | Jamaica | Unknown | 1988 |
| 26 | KY092043 | BTV3 JAM1988/502031 | 3 | Jamaica | Unknown | 1988 |
| 27 | KM099593 | DPP1315 | 1 | Australia | Cattle | 1988 |
| 28 | AJ586661 | IND1988/01 | 1 | India | Unknown | 1988 |
| 29 | AJ631217 | IND1988/02 | 23 | India | Sheep | 1988 |
| 30 | MF384728 | RIVS-178 | 16 | Indonesia | Unknown | 1988 |
| 31 | KF986494 | 185 | 2 | Panama | Cattle | 1989 |
| 32 | KY092058 | BTV14 TOBG1989/502091 | 14 | Trinidad and Tobago | Unknown | 1989 |
| 33 | KY092044 | BTV3 PAN1989/502151 | 3 | Panama | Unknown | 1989 |
| 34 | KY092039 | BTV9 PAN1989/502130 | 9 | Panama | Unknown | 1989 |
| 35 | KM099594 | DPP1523 | 1 | Australia | Cattle | 1989 |
| 36 | MF384690 | E050 | 1 | Australia | Cattle | 1989 |
| 37 | MF384694 | E051 | 1 | Australia | Cattle | 1989 |
| 38 | MF384704 | G082 | 1 | Australia | Cattle | 1989 |
| 39 | MF384751 | RIVS-106 | 23 | Indonesia | Cattle | 1989 |
| 40 | MF384745 | RIVS-66 | 21 | Indonesia | Cattle | 1989 |
| 41 | DQ080912 | BT17-283 | 17 | Puerto Rico | Unknown | 1990 |
| 42 | KY092036 | BTV1 SAL1990/502270 | 1 | Salvador | Unknown | 1990 |
| 43 | KY092057 | BTV14 GUA1990/502230 | 14 | Guatemala | Unknown | 1990 |
| 44 | KY092059 | BTV18 GUA1990/502229 | 18 | Guatemala | Unknown | 1990 |
| 45 | KY092037 | BTV2 PAN1990/185 | 2 | Panama | Unknown | 1990 |
| 46 | KY092041 | BTV3 HON1990/502285 | 3 | Honduras | Unknown | 1990 |
| 47 | MF384746 | D151 | 21 | Indonesia | Unknown | 1990 |
| 48 | MF384729 | RIVS-160 | 16 | Indonesia | Cattle | 1990 |
| 49 | MF384725 | RIVS-53 | 3 | Indonesia | Cattle | 1990 |
| 50 | MF384743 | RIVS-60 | 21 | Indonesia | Cattle | 1990 |
| 51 | MF384744 | RIVS-63 | 21 | Indonesia | Cattle | 1990 |
| 52 | KY092061 | BTV10 GUA1991/502350 | 10 | Guatemala | Unknown | 1991 |
| 53 | KY092038 | BTV9 HON1991/502325 | 9 | Honduras | Unknown | 1991 |
| 54 | MF384747 | RIVS-113 | 21 | Indonesia | Cattle | 1991 |
| 55 | MF384748 | RIVS-137 | 21 | Indonesia | Cattle | 1991 |
| 56 | MF384749 | RIVS-138 | 21 | Indonesia | Cattle | 1991 |
| 57 | MF384750 | RIVS-39 | 23 | Indonesia | Cattle | 1991 |
| 58 | MF384726 | RIVS-74 | 9 | Indonesia | Cattle | 1991 |
| 59 | KY092060 | BTV10 CSTRCA1992/502344 | 10 | Costa Rica | Unknown | 1992 |
| 60 | AJ586659 | IND1992/01 | 1 | India | Sheep | 1992 |
| 61 | AJ586660 | IND1992/02 | 1 | India | Sheep | 1992 |
| 62 | KP696587 | BTV-2/IND1993/01 | 2 | India | Sheep | 1993 |
| 63 | KP339159 | BTV02IND1993 | 2 | India | Sheep | 1993 |
| 64 | KM099595 | DPP2559 | 1 | Australia | Cattle | 1993 |
| 65 | MF384705 | G083 | 1 | Australia | Cattle | 1993 |
| 66 | KM099596 | DPP3072 | 1 | Australia | Cattle | 1994 |
| 67 | KP268779 | IND1994/01 | 2 | India | Unknown | 1994 |
| 68 | JN671911 | BN96/16 | 16 | China | Sheep | 1996 |
| 69 | KM099597 | DPP4032 | 1 | Australia | Cattle | 1996 |
| 70 | MF384721 | E052 | 1 | Australia | Cattle | 1996 |
| 71 | JX560418 | YTS-4 | 4 | China | Cattle | 1996 |
| 72 | KM099598 | DPP4100 | 1 | Australia | Cattle | 1997 |
| 73 | MF384699 | E053 | 1 | Australia | Cattle | 1997 |
| 74 | MF384691 | E054 | 1 | Australia | Cattle | 1997 |
| 75 | MF384706 | G085 | 1 | Australia | Cattle | 1997 |
| 76 | MF384692 | G086 | 1 | Australia | Cattle | 1997 |
| 77 | AJ586728 | IND1997/01 | 23 | India | Unknown | 1997 |
| 78 | JN848764 | SZ97/1 | 1 | China | Sheep | 1997 |
| 79 | MF384730 | G087 | 21 | Australia | Cattle | 1998 |
| 80 | MF384733 | G088 | 21 | Australia | Cattle | 1998 |
| 81 | MF384738 | G089 | 21 | Australia | Cattle | 1998 |
| 82 | MF384739 | G090 | 21 | Australia | Cattle | 1998 |
| 83 | MF384740 | G091 | 21 | Australia | Cattle | 1998 |
| 84 | KP821562 | GRE1998/01 | 9 | Greece | Sheep | 1998 |
| 85 | KP821557 | RSA1998/01 | 8 | South Africa | Goat | 1998 |
| 86 | KP821573 | TUR1998/02 | 9 | Turkey | Sheep | 1998 |
| 87 | MF384700 | 299 | 1 | Australia | Cattle | 1999 |
| 88 | JX024947 | 4/ARG/2001/102 | 4 | Argentina | Cattle | 1999 |
| 89 | JX024952 | 4/ARG/2001/829 | 4 | Argentina | Cattle | 1999 |
| 90 | JX024942 | 4/ARG/2001/99 | 4 | Argentina | Cattle | 1999 |
| 91 | KP821561 | BUL1999/01 | 9 | Bulgaria | Unknown | 1999 |
| 92 | KM099599 | DPP4588 | 1 | Australia | Cattle | 1999 |
| 93 | KM099600 | DPP4690 | 1 | Australia | Cattle | 1999 |
| 94 | KP821563 | GRE1999/06 | 9 | Greece | Sheep | 1999 |
| 95 | KP821471 | GRE1999/13 | 16 | Greece | Sheep | 1999 |
| 96 | KP821534 | GRE1999/24 | 4 | Greece | Sheep | 1999 |
| 97 | KP821535 | GRE1999/26 | 4 | Greece | Culicoides | 1999 |
| 98 | KP821564 | GRE1999/31 | 9 | Greece | Cattle | 1999 |
| 99 | AJ586662 | IND1999/01 | 1 | India | Sheep | 1999 |
| 100 | MF384713 | 705 | 1 | Australia | Cattle | 2000 |
| 101 | JN255877 | BTV-2IT(H) | 2 | Italy | Sheep | 2000 |
| 102 | JN255867 | BTV-2IT(L) | 2 | Italy | Sheep | 2000 |
| 103 | KM053273 | BTV-2IT2000 | 2 | Italy | Sheep | 2000 |
| 104 | JN255917 | BTV-9IT(H) | 9 | Italy | Sheep | 2000 |
| 105 | MF384741 | G092 | 21 | Australia | Cattle | 2000 |
| 106 | MF384712 | G093 | 1 | Australia | Cattle | 2000 |
| 107 | KP821536 | GRE2000/07 | 4 | Greece | Sheep | 2000 |
| 108 | KP821537 | GRE2000/08 | 4 | Greece | Culicoides | 2000 |
| 109 | KP821519 | TUN2000/01 | 2 | Tunisia | Unknown | 2000 |
| 110 | KP821476 | TUR2000/01 | 16 | Turkey | Unknown | 2000 |
| 111 | KP821574 | TUR2000/03 | 9 | Turkey | Unknown | 2000 |
| 112 | MF384707 | 837 | 1 | Australia | Cattle | 2001 |
| 113 | KP821506 | FRA2001/01 | 2 | France | Sheep | 2001 |
| 114 | KP821507 | FRA2001/03 | 2 | France | Sheep | 2001 |
| 115 | KP821508 | FRA2001/06 | 2 | France | Sheep | 2001 |
| 116 | KP821487 | GRE2001/01 | 1 | Greece | Sheep | 2001 |
| 117 | KP821488 | GRE2001/06 | 1 | Greece | Sheep | 2001 |
| 118 | KP821489 | GRE2001/10 | 1 | Greece | Sheep | 2001 |
| 119 | AJ586663 | IND2001/01 | 1 | India | Culicoides | 2001 |
| 120 | KP821509 | ISR2001/17 | 2 | Israel | Sheep | 2001 |
| 121 | KP821473 | ISR2001/18 | 16 | Israel | Sheep | 2001 |
| 122 | KP821566 | KOS2001/01 | 9 | Kosovo | Sheep | 2001 |
| 123 | KP821567 | KOS2001/03 | 9 | Kosovo | Sheep | 2001 |
| 124 | AJ586672 | SAD2001/01 | 2 | Italy | Sheep | 2001 |
| 125 | KP821515 | SAD2001/03 | 2 | Italy | Sheep | 2001 |
| 126 | KP821572 | SER2001/01 | 9 | Serbia | Sheep | 2001 |
| 127 | KP821575 | TUR2001/02 | 9 | Turkey | Sheep | 2001 |
| 128 | MF384693 | 973 | 1 | Australia | Cattle | 2002 |
| 129 | MF384734 | 999 | 21 | Australia | Cattle | 2002 |
| 130 | KP821560 | BOS2002/02 | 9 | Bosnia and Herzegovina | Sheep | 2002 |
| 131 | KM099601 | DPP5775 | 1 | Australia | Cattle | 2002 |
| 132 | KM099602 | DPP5844 | 1 | Australia | Cattle | 2002 |
| 133 | KF387526 | ITL2002 | 16 | Italy | Cattle | 2002 |
| 134 | KP821510 | ITL2002/02 | 2 | Italy | Culicoides | 2002 |
| 135 | AJ586671 | ITL2002/07 | 2 | Italy | Sheep | 2002 |
| 136 | KP821516 | SAD2002/02 | 2 | Italy | Sheep | 2002 |
| 137 | MF384719 | 1121 | 1 | Australia | Cattle | 2003 |
| 138 | KP696567 | BTV-1/IND2003/05 | 1 | India | Sheep | 2003 |
| 139 | KP696577 | BTV-1/IND2003/10 | 1 | India | Sheep | 2003 |
| 140 | KP696597 | BTV-2/IND2003/01 | 2 | India | Sheep | 2003 |
| 141 | KP696607 | BTV-2/IND2003/03 | 2 | India | Sheep | 2003 |
| 142 | JN255897 | BTV-4IT(H) | 4 | Italy | Sheep | 2003 |
| 143 | JN255887 | BTV-4IT(L) | 4 | Italy | Sheep | 2003 |
| 144 | JN255907 | BTV-9IT(L) | 9 | Italy | Sheep | 2003 |
| 145 | KP339179 | BTV09IND2003-M10 | 9 | India | Sheep | 2003 |
| 146 | KP339189 | BTV09IND2003-M11 | 9 | India | Sheep | 2003 |
| 147 | KP339249 | BTV10IND2003K3 | 10 | India | Sheep | 2003 |
| 148 | GU784864 | BTV12/PT/2003 | 12 | Taiwan | Cattle | 2003 |
| 149 | MF384714 | E041 | 1 | Australia | Cattle | 2003 |
| 150 | MF384701 | E074 | 1 | Australia | Cattle | 2003 |
| 151 | KP821531 | FRA2003-03.124 | 4 | France | Unknown | 2003 |
| 152 | KP821532 | FRA2003-200 | 4 | France | Unknown | 2003 |
| 153 | KP821530 | FRA2003/01 | 4 | France | Unknown | 2003 |
| 154 | MF384715 | G095 | 1 | Australia | Cattle | 2003 |
| 155 | AJ783905 | IND2003/02 | 2 | India | Sheep | 2003 |
| 156 | JQ771818 | IND2003/08 | 3 | India | Unknown | 2003 |
| 157 | AJ631215 | ITL2003/01 | 9 | Italy | Sheep | 2003 |
| 158 | AY493690 | KM | 2 | Taiwan | Goat | 2003 |
| 159 | AB686237 | ON-2/C/03 | 9 | Japan | Culicoides | 2003 |
| 160 | KP821549 | SPA2003/03 | 4 | Spain | Sheep | 2003 |
| 161 | KF986517 | USA2003 | 2 | USA | Cattle | 2003 |
| 162 | KX164114 | USA2003/FL 279313 | 14 | USA | Sheep | 2003 |
| 163 | KX164134 | USA2003/FL 280559-3 | 19 | USA | Cattle | 2003 |
| 164 | KX164064 | USA2003/FL 280559-7 | 5 | USA | Cattle | 2003 |
| 165 | KP696675 | BTV-23/IND2004/07 | 23 | India | Unknown | 2004 |
| 166 | KP696685 | BTV-23/IND2004/08 | 23 | India | Unknown | 2004 |
| 167 | KP696695 | BTV-23/IND2004/09 | 23 | India | Unknown | 2004 |
| 168 | KP696617 | BTV-9/IND2004/02 | 9 | India | Unknown | 2004 |
| 169 | KP696627 | BTV-9/IND2004/04 | 9 | India | Unknown | 2004 |
| 170 | KP821470 | COR2004/01 | 16 | France | Sheep | 2004 |
| 171 | KP821467 | CYP2004/01 | 16 | Cyprus | Sheep | 2004 |
| 172 | KP821523 | CYP2004/03 | 4 | Cyprus | Sheep | 2004 |
| 173 | KM099603 | DPP6112 | 1 | Australia | Cattle | 2004 |
| 174 | JQ740776 | IND2004/01 | 10 | India | Unknown | 2004 |
| 175 | KP821543 | MOR2004/02 | 4 | Morocco | Sheep | 2004 |
| 176 | KP821550 | SPA2004/02 | 4 | Spain | Sheep | 2004 |
| 177 | KP696637 | BTV-9/IND2005/01 | 9 | India | Unknown | 2005 |
| 178 | KP696646 | BTV-9/IND2005/02 | 9 | India | Unknown | 2005 |
| 179 | KP696656 | BTV-9/IND2005/03 | 9 | India | Sheep | 2005 |
| 180 | KM099604 | DPP6504 | 1 | Australia | Cattle | 2005 |
| 181 | MF384695 | E042 | 1 | Australia | Cattle | 2005 |
| 182 | MF384708 | E043 | 1 | Australia | Cattle | 2005 |
| 183 | MF384709 | G096 | 1 | Australia | Cattle | 2005 |
| 184 | KP339239 | KMN07/05 | 21 | India | Sheep | 2005 |
| 185 | JQ414051 | M11/MBN/2005 | 9 | India | Sheep | 2005 |
| 186 | KP821517 | SPA2005/01 | 2 | Spain | Cattle | 2005 |
| 187 | KP821551 | SPA2005/02 | 4 | Spain | Cattle | 2005 |
| 188 | MF384720 | 1857 | 1 | Australia | Cattle | 2006 |
| 189 | KP821477 | ALG2006/01 | 1 | Algeria | Sheep | 2006 |
| 190 | KP821478 | ALG2006/04 | 1 | Algeria | Sheep | 2006 |
| 191 | KJ872781 | Ardennes | 8 | France | Sheep | 2006 |
| 192 | JF443160 | BBF | 9 | India | Sheep | 2006 |
| 193 | AM498056 | BTV-8NT (NET2006/04) | 8 | Netherlands | Sheep | 2006 |
| 194 | KP821468 | CYP2006/01 | 16 | Cyprus | Cattle | 2006 |
| 195 | KP821541 | ISR2006/12 | 4 | Israel | Sheep | 2006 |
| 196 | KP821491 | MOR2006/06 | 1 | Morocco | Sheep | 2006 |
| 197 | JX680452 | NET2006/04 | 8 | Netherlands | Cattle | 2006 |
| 198 | KJ736006 | SAD2006 | 1 | Italy | Sheep | 2006 |
| 199 | KF986490 | USA2006 | 2 | USA | Cattle | 2006 |
| 200 | GQ506541 | USA2006/01 | 6 | USA | Cattle | 2006 |
| 201 | MF384716 | 1923 | 1 | Australia | Cattle | 2007 |
| 202 | JX861493 | FRA2007/18 | 1 | France | Sheep | 2007 |
| 203 | KP821486 | GIB2007/06 | 1 | Gibraltar | Sheep | 2007 |
| 204 | JX003692 | Ind-R1-2007 | 9 | India | Sheep | 2007 |
| 205 | KP821490 | LIB2007/06 | 1 | Libya | Sheep | 2007 |
| 206 | KP821492 | MOR2007/01 | 1 | Morocco | Sheep | 2007 |
| 207 | KP821493 | MOR2007/02 | 1 | Morocco | Sheep | 2007 |
| 208 | KP821494 | MOR2007/03 | 1 | Morocco | Sheep | 2007 |
| 209 | KP821495 | MOR2007/04 | 1 | Morocco | Sheep | 2007 |
| 210 | GQ506456 | NET2007/01 | 8 | Netherlands | Cattle | 2007 |
| 211 | KF664118 | SKN7/ABT/HSR | 1 | India | Culicoides | 2007 |
| 212 | KP821500 | SPA2007/04 | 1 | Spain | Cattle | 2007 |
| 213 | KP821501 | SPA2007/05 | 1 | Spain | Cattle | 2007 |
| 214 | KP821505 | TUN2007/01 | 1 | Tunisia | Sheep | 2007 |
| 215 | KP821559 | UKG2007/06 | 8 | UK | Cattle | 2007 |
| 216 | KX164154 | USA2007/FL 520518 | 24 | USA | Deer | 2007 |
| 217 | JQ086296 | v6963 | 7 | Australia | Cattle | 2007 |
| 218 | KP821479 | ALG2008/10 | 1 | Algeria | Sheep | 2008 |
| 219 | KM053263 | BTV-8IT2008 | 8 | Italy | Sheep | 2008 |
| 220 | KM099605 | DPP7137 | 1 | Australia | Cattle | 2008 |
| 221 | KP821481 | FRA2008/21 | 1 | France | Cattle | 2008 |
| 222 | KP821482 | FRA2008/22 | 1 | France | Sheep | 2008 |
| 223 | KP821483 | FRA2008/23 | 1 | France | Cattle | 2008 |
| 224 | JX861503 | FRA2008/24 | 1 | France | Cattle | 2008 |
| 225 | KP821484 | FRA2008/25 | 1 | France | Cattle | 2008 |
| 226 | KP821485 | FRA2008/26 | 1 | France | Cattle | 2008 |
| 227 | KP821462 | FRA2008/27 | 8 | France | Cattle | 2008 |
| 228 | KP821463 | FRA2008/28 | 8 | France | Cattle | 2008 |
| 229 | KP821464 | FRA2008/29 | 8 | France | Cattle | 2008 |
| 230 | KP821555 | GRE2008/01 | 8 | Greece | Sheep | 2008 |
| 231 | KP821472 | GRE2008/10 | 16 | Greece | Sheep | 2008 |
| 232 | KP821542 | ISR2008/02 | 4 | Israel | Cattle | 2008 |
| 233 | KP821474 | ISR2008/03 | 16 | Israel | Goat | 2008 |
| 234 | KP821554 | ISR2008/13 | 8 | Israel | Cattle | 2008 |
| 235 | JX399153 | K23/08 | 1 | India | Sheep | 2008 |
| 236 | KF664108 | K31-08/ABT/HSR | 16 | India | Sheep | 2008 |
| 237 | KP821569 | LIB2008/03 | 9 | Libya | Sheep | 2008 |
| 238 | KP821568 | LIB2008/08 | 9 | Libya | Sheep | 2008 |
| 239 | KU234262 | MKD20/08/Ind | 1 | India | Goat | 2008 |
| 240 | KP821556 | NET2008/03 | 8 | Netherlands | Cattle | 2008 |
| 241 | GQ506477 | NET2008/05 | 6 | Netherlands | Cattle | 2008 |
| 242 | JQ904068 | sheep/08/Ind/ABT/Hisar | 2 | India | Sheep | 2008 |
| 243 | KP821502 | SPA2008/01 | 1 | Spain | Sheep | 2008 |
| 244 | KX164034 | USA2008/AR 566195 | 3 | USA | Deer | 2008 |
| 245 | KX164074 | USA2008/FL 576307 | 9 | USA | Deer | 2008 |
| 246 | JQ086246 | v7291 | 2 | Australia | Cattle | 2008 |
| 247 | JX024957 | 4/ARG/2009 | 4 | Argentina | Cattle | 2009 |
| 248 | MF384696 | E046 | 1 | Australia | Cattle | 2009 |
| 249 | MF384697 | E047 | 1 | Australia | Cattle | 2009 |
| 250 | KP821496 | MOR2009/01 | 1 | Morocco | Sheep | 2009 |
| 251 | KP821498 | MOR2009/04 | 1 | Morocco | Sheep | 2009 |
| 252 | KP821497 | MOR2009/06 | 1 | Morocco | Sheep | 2009 |
| 253 | KP821544 | MOR2009/07 | 4 | Morocco | Sheep | 2009 |
| 254 | KP821545 | MOR2009/08 | 4 | Morocco | Sheep | 2009 |
| 255 | KP821546 | MOR2009/09 | 4 | Morocco | Sheep | 2009 |
| 256 | KP821547 | MOR2009/10 | 4 | Morocco | Sheep | 2009 |
| 257 | KP821503 | SPA2009/01 | 1 | Spain | Cattle | 2009 |
| 258 | MF384723 | 2010-21 | 2 | Australia | Cattle | 2010 |
| 259 | MF384724 | 2010-26 | 2 | Australia | Cattle | 2010 |
| 260 | MF384722 | 2010-90 | 2 | Australia | Cattle | 2010 |
| 261 | JX024962 | 4/ARG/2010 | 4 | Argentina | Cattle | 2010 |
| 262 | JQ972856 | BTV-11_DE | 11 | Germany | Cattle | 2010 |
| 263 | JQ972866 | BTV-11_MQ | 11 | Martinique | Cattle | 2010 |
| 264 | KP339139 | BTV01IND2010-KRM07 | 1 | India | Sheep | 2010 |
| 265 | KP339149 | BTV01IND2010-VC12 | 1 | India | Sheep | 2010 |
| 266 | KP339169 | BTV02IND2010-KRM08 | 2 | India | Sheep | 2010 |
| 267 | KP339199 | BTV16IND2010-AP04 | 16 | India | Sheep | 2010 |
| 268 | KP339209 | BTV16IND2010-AP06 | 16 | India | Sheep | 2010 |
| 269 | KP339229 | BTV16IND2010-VC07 | 16 | India | Sheep | 2010 |
| 270 | JQ240326 | Cooktown | 2 | Australia | Cattle | 2010 |
| 271 | KP821469 | CYP2010/03 | 16 | Cyprus | Cattle | 2010 |
| 272 | KM099606 | DPP8086 | 1 | Australia | Cattle | 2010 |
| 273 | KM099607 | DPP8304 | 1 | Australia | Cattle | 2010 |
| 274 | MF384710 | G099 | 1 | Australia | Cattle | 2010 |
| 275 | MF384731 | G100 | 21 | Australia | Cattle | 2010 |
| 276 | MF384717 | G101 | 1 | Australia | Cattle | 2010 |
| 277 | JQ924825 | IND/Goat/2010/16/HSR | 16 | India | Goat | 2010 |
| 278 | JX007927 | IND2010/cattle/16 | 16 | India | Cattle | 2010 |
| 279 | KJ577119 | SAD2010 | 1 | Italy | Sheep | 2010 |
| 280 | KP821552 | SPA2010/01 | 4 | Spain | Cattle | 2010 |
| 281 | JQ822253 | USA2010 | 2 | USA | Cattle | 2010 |
| 282 | MF384742 | v8184 | 21 | Australia | Cattle | 2010 |
| 283 | MF384718 | 2011-11 | 1 | Australia | Cattle | 2011 |
| 284 | MF384711 | 2011-3 | 1 | Australia | Cattle | 2011 |
| 285 | KY049848 | BTV-1/11-01 (4074) | 1 | Guyane | Cattle | 2011 |
| 286 | KP339219 | BTV16IND2011-NR82 | 16 | India | Sheep | 2011 |
| 287 | KP821524 | CYP2011/01 | 4 | Cyprus | Sheep | 2011 |
| 288 | KP821525 | CYP2011/02 | 4 | Cyprus | Sheep | 2011 |
| 289 | KP821526 | CYP2011/03 | 4 | Cyprus | Sheep | 2011 |
| 290 | KP821527 | CYP2011/04 | 4 | Cyprus | Sheep | 2011 |
| 291 | KP821528 | CYP2011/05 | 4 | Cyprus | Sheep | 2011 |
| 292 | KF664138 | G53/ABT/HSR | 16 | India | Goat | 2011 |
| 293 | KP821578 | RUS2011/01 | 14 | Russia | Cattle | 2011 |
| 294 | KJ577109 | TUN2011 | 1 | Tunisia | Sheep | 2011 |
| 295 | KM580451 | USA2011/TX 11-56803-18 | 11 | USA | Deer | 2011 |
| 296 | KM580422 | USA2011/TX 11-56803-3 | 11 | USA | Deer | 2011 |
| 297 | KM580424 | USA2011/TX 11-56803-5 | 11 | USA | Deer | 2011 |
| 298 | KM580434 | USA2011/TX 11-56803-9 | 11 | USA | Deer | 2011 |
| 299 | KM580459 | USA2011/TX 128184-11 | 11 | USA | Dog | 2011 |
| 300 | MF384698 | 2012-12 | 1 | Australia | Cattle | 2012 |
| 301 | MF384702 | 2012-26 | 1 | Australia | Cattle | 2012 |
| 302 | MF384703 | 2012-29 | 1 | Australia | Cattle | 2012 |
| 303 | MF384732 | 2012-31 | 21 | Australia | Cattle | 2012 |
| 304 | MF384735 | 2012-33 | 21 | Australia | Cattle | 2012 |
| 305 | MF384736 | 2012-44 | 21 | Australia | Cattle | 2012 |
| 306 | KP821538 | GRE2012/01 | 4 | Greece | Sheep | 2012 |
| 307 | KP821539 | GRE2012/02 | 4 | Greece | Sheep | 2012 |
| 308 | KP821540 | GRE2012/03 | 4 | Greece | Sheep | 2012 |
| 309 | KP821577 | POL2012/01 | 14 | Poland | Cattle | 2012 |
| 310 | KJ577099 | SAD2012 | 1 | Italy | Sheep | 2012 |
| 311 | KP821579 | SPA2012/01 | 14 | Spain | Cattle | 2012 |
| 312 | KM580471 | USA2012/KS 120659-12 | 11 | USA | Dog | 2012 |
| 313 | KX164044 | USA2012/SD 12-035694 | 3 | USA | Deer | 2012 |
| 314 | KJ019210 | BTV-1 SAD2013 | 1 | Italy | Sheep | 2013 |
| 315 | KJ577129 | LAZ2013 | 1 | Italy | Sheep | 2013 |
| 316 | KJ661734 | SIC2013 | 1 | Italy | Unknown | 2013 |
| 317 | KX164104 | USA2013/CA 13-034210 | 13 | USA | Deer | 2013 |
| 318 | KM580480 | USA2013/FL 13-037190 | 11 | USA | Deer | 2013 |
| 319 | KM580485 | USA2013/WA 13-031503 | 11 | USA | Llama | 2013 |
| 320 | LN713675 | 379 | 27 | France | Goat | 2014 |
| 321 | KX599364 | 17/BRA/2014/73 | 17 | Brazil | Sheep | 2014 |
| 322 | KU760992 | BTV-27/FRA2014/v02 | 27 | France | Goat | 2014 |
| 323 | KU761002 | BTV-27/FRA2014/v03 | 27 | France | Goat | 2014 |
| 324 | KP268819 | BTV4-HUN2014 | 4 | Hungary | Cattle | 2014 |
| 325 | KT002583 | GDST008 | 7 | China | Cattle | 2014 |
| 326 | KX302639 | IND2014/01 | 16 | India | Sheep | 2014 |
| 327 | KX164124 | USA2014/FL 15-008010 | 18 | USA | Deer | 2014 |
| 328 | KX695175 | V196/XJ/2014 | ? | China | Goat | 2014 |
| 329 | KR061883 | XJ1407 | ? | China | Goat | 2014 |
| 330 | KU569995 | 15-01 | 8 | France | Sheep | 2015 |
| 331 | MG206082 | 5149E | 21 | China | Cattle | 2015 |
| 332 | KY049858 | BTV-1/15.01 (5370) | 1 | Guyane | Cattle | 2015 |
| 333 | KY049876 | BTV-13/15-01 (6) | 13 | Ecuador | Cattle | 2015 |
| 334 | KY049885 | BTV-18/15-01 (58) | 18 | Ecuador | Cattle | 2015 |
| 335 | KY049867 | BTV-9/15-01 (7) | 9 | Ecuador | Cattle | 2015 |
| 336 | KX164054 | USA2015/TX 15-029176 | 3 | USA | Cattle | 2015 |
| 337 | KY654333 | BTV-4/16-03 | 4 | France | Sheep | 2016 |

**Table S3. Summary profile of BTV segment 10 gene sequences isolated worldwide between 1958 and 2016 (n = 389).**

| ***Continent*** | **Country/Host** | **Unknown** | **Cattle** | **Culicoides** | **Deer** | **Goat** | **Sheep** | **Total** |
| --- | --- | --- | --- | --- | --- | --- | --- | --- |
| ***Africa*** |  | 11 | 1 | 1 |  | 1 | 18 | 32 |
|  | **Algeria** | **-** | - | - | - | - | 2 | 2 |
|  | **Cameroon** | 1 | - | - | - | - | 1 | 2 |
|  | **Egypt** | 1 | - | - | - | - | - | 1 |
|  | **Libya** | **-** | - | - | - | - | 3 | 3 |
|  | **Morocco** | **-** | - | - | - | - | 9 | 9 |
|  | **Nigeria** | 1 | - | - | - | - | - | 1 |
|  | **South Africa** | 6 | - | - | - | 1 | 1 | 8 |
|  | **Sudan** | 1 | 1 | 1 | - | - | - | 3 |
|  | **Tunisia** | 1 | - | - | - | - | 2 | 3 |
| ***East Asia*** |  | 10 | 17 | 2 |  | 6 | 41 | 76 |
|  | **China** | **-** | 3 | - | - | 2 | 2 | 7 |
|  | **India** | 10 | 1 | 1 | - | 3 | 39 | 54 |
|  | **Indonesia** | **-** | 12 | - | - | - | - | 12 |
|  | **Japan** | **-** | - | 1 | - | - | - | 1 |
|  | **Taiwan** | **-** | 1 | - | - | 1 | - | 2 |
| ***Europe*** |  | 5 | 20 | 1 |  | 4 | 42 | 72 |
|  | **Bosnia and Herzegovina** | **-** | - | - | - | - | 1 | 1 |
|  | **Bulgaria** | 1 | - | - | - | - | - | 1 |
|  | **France** | 3 | 3 | - | - | 2 | 8 | 16 |
|  | **Germany** | **-** | 1 | - | - | - | - | 1 |
|  | **Gibraltar** | **-** | - | - | - | - | 1 | 1 |
|  | **Greece** | 1 | 2 | 1 | - | 1 | 11 | 16 |
|  | **Hungary** | **-** | 1 | - | - | - | - | 1 |
|  | **Italy** | **-** | 1 | - | - | 1 | 18 | 20 |
|  | **Kosovo** | **-** | - | - | - | - | 2 | 2 |
|  | **Netherlands** | **-** | 4 | - | - | - | - | 4 |
|  | **Portugal** | **-** | 1 | - | - | - | 1 | 2 |
|  | **Russia** | **-** | 1 | - | - | - | - | 1 |
|  | **Spain** | **-** | 5 | - | - | - | - | 5 |
|  | **United Kingdom** | **-** | 1 | - | - | - | - | 1 |
| ***Middle America*** |  | 18 | 5 | - | - | - | - | 23 |
|  | **Barbados** | 1 | - | - | - | - | - | 1 |
|  | **Costa Rica** | 2 | - | - | - | - | - | 2 |
|  | **Dominican Republic** | 2 | - | - | - | - | - | 2 |
|  | **Guatemala** | 2 | - | - | - | - | - | 2 |
|  | **Honduras** | 4 | - | - | - | - | - | 4 |
|  | **Jamaica** | 2 | - | - | - | - | - | 2 |
|  | **Martinique** |  | 4 | - | - | - | - | 4 |
|  | **Panama** | 2 | 1 | - | - | - | - | 3 |
|  | **Puerto Rico** | 2 | - | - | - | - | - | 2 |
|  | **Salvador** | 1 | - | - | - | - | - | 1 |
| ***North America*** |  | 2 | 24 | - | 28 | 1 | 12 | 67 |
|  | **USA** | 2 | 24 | - | 28 | 1 | 12 | 67 |
| ***Australia*** |  | - | 50 | 1 | - | - | - | 51 |
|  | **Australia** | **-** | 50 | 1 | - | - | - | 51 |
| ***South America*** |  | 2 | 10 | - | - | - | 1 | 13 |
|  | **Argentina** | **-** | 5 | - | - | - | - | 5 |
|  | **Brazil** | - | - | - | - | - | 1 | 1 |
|  | **Ecuador** | **-** | 3 | - | - | - | - | 3 |
|  | **Guyane** | **-** | 2 | - | - | - | - | 2 |
|  | **Trinidad and Tobago** | 2 | - | - | - | - | - | 2 |
| ***West Asia*** |  | 8 | 11 | - | - | 1 | 35 | 55 |
|  | **Cyprus** | 1 | 2 | - | - | - | 2 | 5 |
|  | **Israel** | 2 | 9 | - | - | 1 | 15 | 27 |
|  | **Turkey** | 5 | - | - | - | - | 18 | 23 |
| **Total** |  | 56 | 138 | 5 | 28 | 13 | 149 | 389 |

**Table S4. Summary profile of BTV segment 6 gene sequences isolated worldwide between 1958 and 2016 (n = 389).**

| ***Continent*** | **Country/Host** | Unknown | Cattle | Culicoides | Deer | Dog | Goat | Llama | Sheep | **Total** |
| --- | --- | --- | --- | --- | --- | --- | --- | --- | --- | --- |
| ***Africa*** |  | 3 | 12 |  | 4 | 1 | 1 |  | 9 | **30** |
|  | **Algeria** | 2 | 1 |  |  |  |  |  |  | 3 |
|  | **Cameroon** |  | 1 |  |  |  |  |  | 1 | 2 |
|  | **Egypt** | 1 |  |  |  |  |  |  |  | 1 |
|  | **Libya** |  | 1 |  |  |  | 1 |  | 1 | 3 |
|  | **Morocco** |  | 4 |  | 2 |  |  |  | 7 | 13 |
|  | **Nigeria** |  | 1 |  |  |  |  |  |  | 1 |
|  | **South Africa** |  | 1 |  |  |  |  |  |  | 1 |
|  | **Sudan** |  |  |  | 2 | 1 |  |  |  | 3 |
|  | **Tunisia** |  | 3 |  |  |  |  |  |  | 3 |
| ***East Asia*** |  | 6 | 38 | 1 | 1 |  | 2 |  | 26 | **74** |
|  | **China** |  | 5 |  |  |  |  |  | 3 | 8 |
|  | **India** | 6 | 25 | 1 | 1 |  | 1 |  | 16 | 50 |
|  | **Indonesia** |  | 6 |  |  |  |  |  | 7 | 13 |
|  | **Japan** |  | 1 |  |  |  |  |  |  | 1 |
|  | **Taiwan** |  | 1 |  |  |  | 1 |  |  | 2 |
| ***Europe*** |  | 10 | 37 | 2 | 2 |  | 3 |  | 33 | **87** |
|  | **BH** |  | 1 |  |  |  |  |  |  | 1 |
|  | **Bulgaria** |  | 1 |  |  |  |  |  |  | 1 |
|  | **France** | 8 | 6 | 2 |  |  | 1 |  | 6 | 23 |
|  | **Germany** | 1 |  |  |  |  |  |  |  | 1 |
|  | **Gibraltar** |  | 1 |  |  |  |  |  |  | 1 |
|  | **Greece** |  | 7 |  |  |  |  |  | 10 | 17 |
|  | **Hungary** |  |  |  |  |  |  |  | 1 | 1 |
|  | **Italy** | 1 | 14 |  |  |  | 1 |  | 5 | 21 |
|  | **Kosovo** |  |  |  |  |  |  |  | 2 | 2 |
|  | **Netherlands** |  | 3 |  |  |  |  |  | 2 | 5 |
|  | **Poland** |  | 1 |  |  |  |  |  |  | 1 |
|  | **Russia** |  | 1 |  |  |  |  |  |  | 1 |
|  | **Serbia** |  | 1 |  |  |  |  |  |  | 1 |
|  | **Spain** |  | 1 |  | 2 |  | 1 |  | 6 | 10 |
|  | **UK** |  |  |  |  |  |  |  | 1 | 1 |
| ***Middle America*** |  | 4 | 8 | 1 |  |  |  |  | 3 | **16** |
|  | **Barbados** |  | 1 |  |  |  |  |  |  | 1 |
|  | **CR** |  |  |  |  |  |  |  | 1 | 1 |
|  | **Guatemala** |  | 3 |  |  |  |  |  |  | 3 |
|  | **Honduras** | 1 |  |  |  |  |  |  | 1 | 2 |
|  | **Jamaica** |  | 2 |  |  |  |  |  |  | 2 |
|  | **Martinique** | 1 |  |  |  |  |  |  |  | 1 |
|  | **Panama** | 1 | 1 | 1 |  |  |  |  | 1 | 4 |
|  | **PR** |  | 1 |  |  |  |  |  |  | 1 |
|  | **Salvador** | 1 |  |  |  |  |  |  |  | 1 |
| ***North America*** |  | 1 | 4 |  | 4 | 1 | 5 | 1 | 6 | **22** |
|  | **USA** | 1 | 4 |  | 4 | 1 | 5 | 1 | 6 | 22 |
| ***Australia*** |  | 10 | 28 | 3 |  |  |  |  | 34 | **75** |
|  | **Australia** | 10 | 28 | 3 |  |  |  |  | 34 | 75 |
| ***South America*** |  | 7 | 4 |  |  |  |  |  | 1 | **12** |
|  | **Argentina** | 4 | 1 |  |  |  |  |  |  | 5 |
|  | **Brazil** | 1 |  |  |  |  |  |  |  | 1 |
|  | **Ecuador** | 2 |  |  |  |  |  |  | 1 | 3 |
|  | **Guyane** |  | 2 |  |  |  |  |  |  | 2 |
|  | **TT** |  | 1 |  |  |  |  |  |  | 1 |
| ***West Asia*** |  | 3 | 10 | 1 |  |  |  |  | 7 | **21** |
|  | **Cyprus** | 3 | 3 | 1 |  |  |  |  | 4 | 11 |
|  | **Israel** |  | 5 |  |  |  |  |  | 1 | 6 |
|  | **Turkey** |  | 2 |  |  |  |  |  | 2 | 4 |
| **Total** |  | 44 | 141 | 8 | 11 | 2 | 11 | 1 | 119 | 337 |

**Table S5. Bayes factor (BF) comparisons of segment 10 demographic phylodynamic models using path-sampling (PS) and stepping-stone (SS) methods.** BFs based on the PS marginal likelihood estimates are on the upper off-diagonal of the table, while BFs based on SS marginal likelihood estimates are on the lower off-diagonal of thetable. Best fitting models have been boldfaced.

|  |  |  |  | **Bayes Factor** |  |  |  | |
| --- | --- | --- | --- | --- | --- | --- | --- | --- |
| **Model** | UCED+CP | UCED+EG | UCED+EGx | UCED+SG | UCLN+CP | UCLN+EG | UCLN+SG |  |
| UCED^a^+CP^b^ | –– | -3 | 615 | -27 | -32 | -50 | -22 |  |
| UCED+EG^c^ | -44 | –– | 618 | -24 | -29 | -47 | -19 |  |
| UCED+EGx^d^ | -643 | -599 | –– | -642 | -647 | -665 | -637 |  |
| UCED+SG^f^ | -13 | 31 | 630 | –– | -5 | -23 | 5 |  |
| UCLN^g^+CP | -9 | 35 | 634 | 4 | –– | -18 | 10 |  |
| **UCLN+EG** | **5** | **39** | **638** | **8** | **4** | **––** | **28** |  |
| UCLN+EGx | -676 | -632 | -33 | -663 | -667 | -671 | -672 |  |
| UCLN+SG | –– | -3 | 615 | -27 | -32 | -50 | -22 |  |

^a^Uncorrelated relaxed clock with exponential distribution

^b^Constant population size coalescent model

^c^Expansion population size coalescent model

^d^Exponential population size coalescent model

^f^Bayesian skylgrid coalescent model

^g^Uncorrelated relaxed clock with log-normal distribution

**Table S6. Bayes factor (BF) comparisons of segment 6 demographic phylodynamic models using path-sampling (PS) and stepping-stone (SS) methods.** BFs based on the PS marginal likelihood estimates are on the upper off-diagonal of the table, while BFs based on SS marginal likelihood estimates are on the lower off-diagonal of thetable. Best fitting models have been boldfaced.

|  |  |  |  | **Bayes Factor** |  |  |  | |
| --- | --- | --- | --- | --- | --- | --- | --- | --- |
| **Model** | UCED+CP | UCED+EG | UCED+EGx | UCED+SG | UCLN+CP | UCLN+EGx | | UCLN+SG |
| UCED^a^+CP^b^ | 1 | 2 | 3 | 4 | 5 | 7 | | 8 |
| UCED+EG^c^ | –– | -10 | 765 | -167 | 85 | 659 | | -165 |
| UCED+EGx^d^ | -1 | –– | 775 | -157 | 95 | 669 | | -155 |
| UCED+SG^f^ | -743 | -742 | –– | -932 | -680 | -106 | | -930 |
| UCLN^g^+CP | 176 | 177 | 919 | –– | 252 | 826 | | 2 |
| **UCLN+EG** | **359** | **360** | **1102** | **183** | **429** | **1019** | | **195** |
| UCLN+EGx | -70 | -69 | 673 | -246 | –– | 574 | | -250 |
| UCLN+SG | -628 | -627 | 115 | -804 | -558 | –– | | -824 |

^a^Uncorrelated relaxed clock with exponential distribution

^b^Constant population size coalescent model

^c^Expansion population size coalescent model

^d^Exponential population size coalescent model

^f^Bayesian skylgrid coalescent model

^g^Uncorrelated relaxed clock with log-normal distribution

**Table S7.** **Association indexes and parsimony scores for the selected discrete trait phylodynamic models.**

| **Discrete trait** |  | **AI^1^** | **95% CI** | **P-value** | **PS^2^** | **95% CI** | **p-value** |
| --- | --- | --- | --- | --- | --- | --- | --- |
| *Segment 10* | | | | | | | |
| Continent | Observed | 34.2 | (33.7, 36.8) | <0.001 | 299.1 | (295.0, 302.0) | <0.001 |
|  | Null | 39.6 | (36.1, 42.6) |  | 318.5 | (310.4, 324.1) |  |
| Country | Observed | 36.7 | (35.1, 38.1) | < 0.001 | 272.7 | (269.0, 277.0) | < 0.001 |
|  | Null | 42.7 | (41.3, 44.1) |  | 303.0 | (296.1, 309.8) |  |
| Host | Observed | 30.2 | (28.7, 31.8) | 0.004 | 205.5 | (201.0, 209.0) | 0.005 |
|  | Null | 33.6 | (31.5, 35.6) |  | 218.7 | (210.4, 227.1) |  |
| *Segment 6* | | | | | | | |
| Continent | Observed | 41.3 | (39.3, 44.8) | <0.001 | 322.1 | (316.3, 326.1) | <0.001 |
|  | Null | 47.6 | (45.1, 49.1) |  | 333.1 | (328.0, 337.0) |  |
| Country | Observed | 51.4 | (48.1, 54.2) | < 0.001 | 301.7 | (290.0, 306.0) | < 0.001 |
|  | Null | 59.1 | (55.3, 61.4) |  | 312.0 | (307.3, 320.7) |  |
| Host | Observed | 30.1 | (28.9, 32.1) | 0.010 | 193.5 | (185.0, 195.0) | 0.030 |
|  | Null | 34.2 | (32.9, 36.1) |  | 196.1 | (195.3, 199.1) |  |

^1^Association Index (AI)

^2^Parsimony Score (PS)
